# Germ cell-intrinsic effects of sex chromosomes on early oocyte differentiation in mice

**DOI:** 10.1371/journal.pgen.1008676

**Published:** 2020-03-26

**Authors:** Norio Hamada, Nobuhiko Hamazaki, So Shimamoto, Orie Hikabe, Go Nagamatsu, Yuki Takada, Kiyoko Kato, Katsuhiko Hayashi

**Affiliations:** 1 Department of Stem Cell Biology and Medicine, Graduate School of Medical Sciences, Kyushu University, Maidashi, Higashi-ku, Fukuoka, Japan; 2 Department of Obstetrics and Gynecology, Graduate School of Medical Sciences, Kyushu University, Maidashi, Higashi-ku, Fukuoka, Japan; Cornell University, UNITED STATES

## Abstract

A set of sex chromosomes is required for gametogenesis in both males and females, as represented by sex chromosome disorders causing agametic phenotypes. Although studies using model animals have investigated the functional requirement of sex chromosomes, involvement of these chromosomes in gametogenesis remains elusive. Here, we elicit a germ cell-intrinsic effect of sex chromosomes on oogenesis, using a novel culture system in which oocytes were induced from embryonic stem cells (ESCs) harboring XX, XO or XY. In the culture system, oogenesis using XO and XY ESCs was severely disturbed, with XY ESCs being more strongly affected. The culture system revealed multiple defects in the oogenesis of XO and XY ESCs, such as delayed meiotic entry and progression, and mispairing of the homologous chromosomes. Interestingly, *Eif2s3y*, a Y-linked gene that promotes proliferation of spermatogonia, had an inhibitory effect on oogenesis. This led us to the concept that male and female gametogenesis appear to be in mutual conflict at an early stage. This study provides a deeper understanding of oogenesis under a sex-reversal condition.

## Author summary

Oogenesis is a highly sex-dependent differentiation process that commences at the mid-gestation stage. Oogenesis is severely disturbed in XO and XY females, although the mechanisms underlying the disturbance are still elusive. In this study, we subjected XO and XY ES cells to an *in vitro* culture system, in which female (XX) ES cells differentiate into functional oocytes. Our results showed that oogenesis from XO and XY ES cells was severely disturbed due to defective processes such as delayed meiotic initiation/progression, aberrant gene expression and mispairing of homologous chromosomes. In addition, our results demonstrate that a Y-linked gene, *Eif2s3y* that promotes spermatogenesis, has conversely a negative impact on oogenesis. This study provides a culture system that serves as a useful model of sex reversal and contributes the concept of a substantial germ cell-intrinsic conflict between male and female gametogenesis. Further study using the culture system will reveal molecular insights into infertility with sex chromosome disorder(s).

## Introduction

Oogenesis is a unique sequence of differentiation processes that finally confers totipotency to eggs. The process is strictly regulated in a sex-dependent manner in mammals. In mice, primordial germ cells (PGCs) arise from the pluripotent epiblast and migrate into embryonic gonads, which eventually become either ovaries or testes [1, 2]. The sex difference is not obvious in nascent and migratory PGCs but becomes evident after reaching the gonad stage: PGCs enter meiosis in females, thereby becoming primary oocytes, whereas they continue to proliferate and are eventually arrested at the G0/G1 phase in males [3]. The sex-dependent differentiation is dominated by gonadal somatic cells whose sex differentiation precedes germ cells [4]. In females, meiosis is induced by retinoic acid (RA) and BMP2, which are produced by mesonephros and gonadal somatic cells, respectively [5, 6]. Conversely, in the male gonads, RA is degraded by CYP26B1 and BMP2 is not expressed, and therefore the inducers of meiotic entry are absent [5, 7]. It has been shown that male PGCs enter meiosis when cultured with female gonadal somatic cells [3] or upon stimulation with RA and BMP2 [8], demonstrating that the initial sex differentiation of germ cells is indeed controlled by a somatic environment. However, it appears that female PGCs cannot differentiate into sperm, and the ability of male PGCs to differentiate into functional eggs is compromised. These phenomena have been well characterized by experiments using sex-reversal mouse models. For example, XX males showed defective differentiation/proliferation of spermatogonia and lack of spermatogenesis [9, 10], while XY females exhibited a reduction in oocyte number in the ovary and compromised fertility [11–13].

Several potential causes of the defective gametogenesis of “sex-mismatched” germ cells have been proposed. In the case of defective oogenesis in XY females, a half dosage of X chromosomes, a heterologous pairing of the sex chromosome and resulting asynapsis during meiosis, and a negative impact of Y-linked gene(s) are the main postulated mechanisms. It is known that one of the X chromosomes is inactivated in nascent PGCs, but reactivated prior to meiotic entry. Therefore, XY oocytes are essentially insufficient of transcripts from the X chromosome. During meiosis, all homologous chromosomes align by the pachytene stage. In spermatogenesis, a unique regulatory action takes place between X and Y chromosomes via a structure known as the XY body, in which the transcriptional activity of the sex chromosome is silenced in the spermatocytes [14]. This silencing, called meiotic sex chromosome inactivation (MSCI), is required for meiotic progression. However, the XY body is rarely formed in XY oocytes, suggesting that there is a difference in regulation of the heterologous chromosome in oocytes. It is known that Y-linked genes play a key role in spermatogenesis. Considering that the Y chromosome appears only in spermatogenesis but not in oogenesis, it is possible that genes having a negative role on oogenesis are accommodated in the Y chromosome. Indeed, it has been reported that a Y-linked gene, *Zfy2*, disturbed meiotic progression in oocytes [15]. Although several possible functions have been proposed, the role of sex chromosomes in oogenesis has not been fully elucidated. This is partially due to the use of sex-reversal mice, the phenotypes of which vary depending on their genetic background and the cause of sex-reversal [13]. In addition, most of the strains have mutations or deletions both in germ cells and somatic cells, which makes it impossible to entirely isolate a germ cell-intrinsic function of genes/chromosomes on oogenesis. Furthermore, it is difficult to manipulate genes in the sex reversal strains due to the compromised fertility. To overcome these problems, there is need of an alternative approach to address the role of sex chromosomes in oogenesis.

Recently, we reported that oogenesis can be reconstituted in culture using pluripotent stem cells such as embryonic stem cells (ESCs) and induced pluripotent stem cells (iPSCs). In the reported culture system, PGC-like cells (PGCLCs) derived from ESCs/iPSCs were aggregated with female gonadal somatic cells of embryonic day (E) 12.5 embryos [16], thereafter called reconstituted ovary (rOvary). Notably, the oogenesis of this culture system, named *in vitro* differentiation (IVDi), largely reproduced that of *in vivo* differentiation in terms of gene expression, morphology and functionality: eggs produced in the culture system gave rise to live pups. This culture system seems advantageous from the following standpoints: (1) the functions of germ and somatic cells can be separately evaluated in the reconstitution system; (2) cells without mutation/deletion can be used; and (3) it is easy to evaluate gene function by manipulating the gene(s) in ESCs/iPSCs. Therefore, in this study, we used this novel culture system as a tool to gain insight into the germ cell-intrinsic role of sex chromosomes on oogenesis.

## Results

### Disrupted oogenesis from XO and XY ESCs

**Fig 1:**
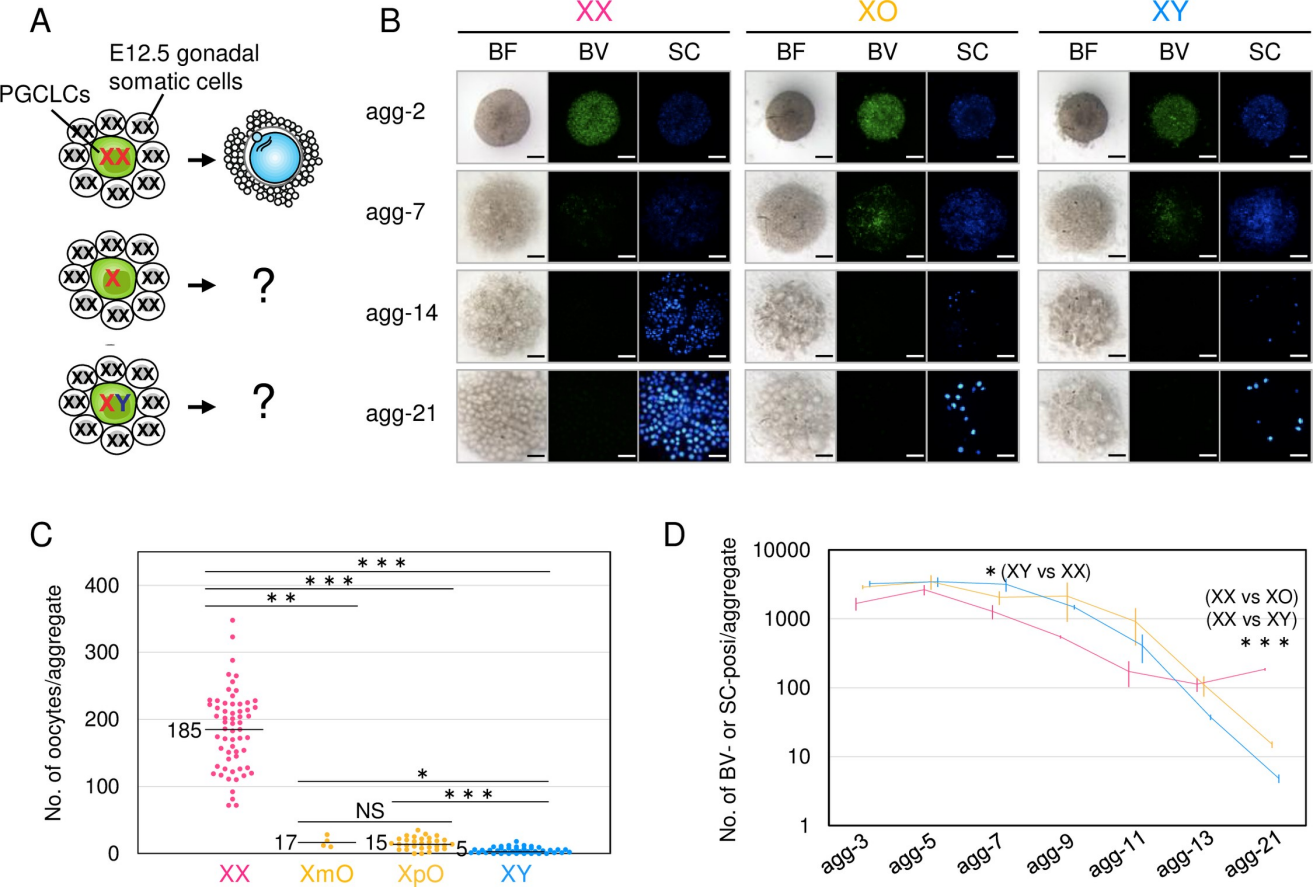
Formation of XX, XO and XY oocytes in culture. (**A**) Schematic illustration of the experimental design. PGCLCs derived from XX, XO and XY ESCs were reaggregated with E12.5 female (XX) gonadal somatic cells. (**B**) Oocyte differentiation from XX, XO and XY ESCs in culture. Images are rOvaries at the days indicated. Blimp1-mVenus (BV) is a marker of PGC(LC)s and stella-CFP (SC) is a marker of PGC(LC)s and oocytes. BF, bright field. Scale bars, 200 μm. (**C**) The number of oocytes formed in culture. Each dot indicates the number of oocytes formed in one rOvary. The numbers in the graph indicate the average number of oocytes/rOvary formed in each genotype. XpO and XmO indicate cells having a paternal and maternal X chromosome, respectively. (**D**) Time-course of the number of PGCLCs/oocytes. The average number of oocytes at the days indicated and SD are shown. The data was compiled from at least three independent experiments. *P* values were calculated by Steel Dwass test. ***P<0.001, **P<0.01, *P<0.05; NS, not significant.

First, we examined oocyte differentiation from ESCs harboring XX, XY or a single X chromosome (XO) in rOvaries composed of E12.5 female gonadal somatic cells (Fig 1A). XO ESCs were generated from XX ESCs by a targeted deletion of one X chromosome (S1A, S1B and S1C Fig) based on a previous report [17]. XO was confirmed by loss of the punctate H3K27me3 signal (which marks an inactive X chromosome) upon differentiation (S1D Fig); polymorphism between the X chromosomes (S1E Fig); and karyotyping (S1F Fig). The polymorphism analyses distinguished XO harboring the maternal X chromosome (XmO) from XO harboring the paternal X chromosome (XpO) (S1E Fig). Using these ESC lines, PGCLCs were induced and then reaggregated with somatic cells of E12.5 female gonads. In the induction, a majority (71.5%) of PGCLCs derived from XX ESCs maintained two X chromosomes (S1G Fig). All PGCLCs were well mingled with gonadal somatic cells (Fig 1B), as the expressions of *Blimp1*-mVenus (BV), a reporter gene of PGCs, and *stella*-ECFP (SC), a reporter gene of PGCs and oocytes, were clearly observed in aggregates at 2 days of culture (agg-2). As expected, in XX PGCLC, BV was downregulated from 7 days of culture onward (agg-7) (Fig 1B), during which period PGCLCs entered meiosis [16]. At the same time, some degree of BV expression remained in XO and XY PGCLCs/oocytes. At 14 days of culture (agg-14), a number of small oocytes were formed in aggregates with XX PGCLCs, while few were observed with XO and XY PGCLCs (Fig 1B). At 21 days of culture (agg-21), some SC-positive XO and XY oocytes appeared, but the numbers of these cells were ~12-fold lower than the number of XX oocytes (Fig 1B and 1C). It was also noteworthy that the number of XY oocytes was significantly lower than that of XO oocytes (Fig 1C). The reduction of XY oocytes was confirmed by rOvaries using PGCs in vivo: E11.5 XY PGCs rarely formed oocytes under the female somatic cell environment, whereas E11.5 XX PGCs formed a certain number of oocytes under the same environment (S2A and S2B Fig). The numbers of XmO and XpO oocytes were not significantly different (Fig 1C), suggesting that there is no functional difference between maternal and paternal X chromosomes; therefore, the subsequent analyses were performed using XpO ESCs. The number of PGCLCs/oocytes was comparable at the initial stage until agg-7, and it was slightly higher in XO and XY than in XX PGCLCs/oocytes between agg-7 and -11 (Fig 1D). However, the numbers of XO and XY PGCLCs/oocytes began to steadily decline after agg-11. These results demonstrated that the sex chromosome composition in germ cells has an impact on oogenesis *in vitro* even under the same somatic cell environment.

### Transcriptome analysis of ESC-derived XO and XY oocytes

**Fig 2:**
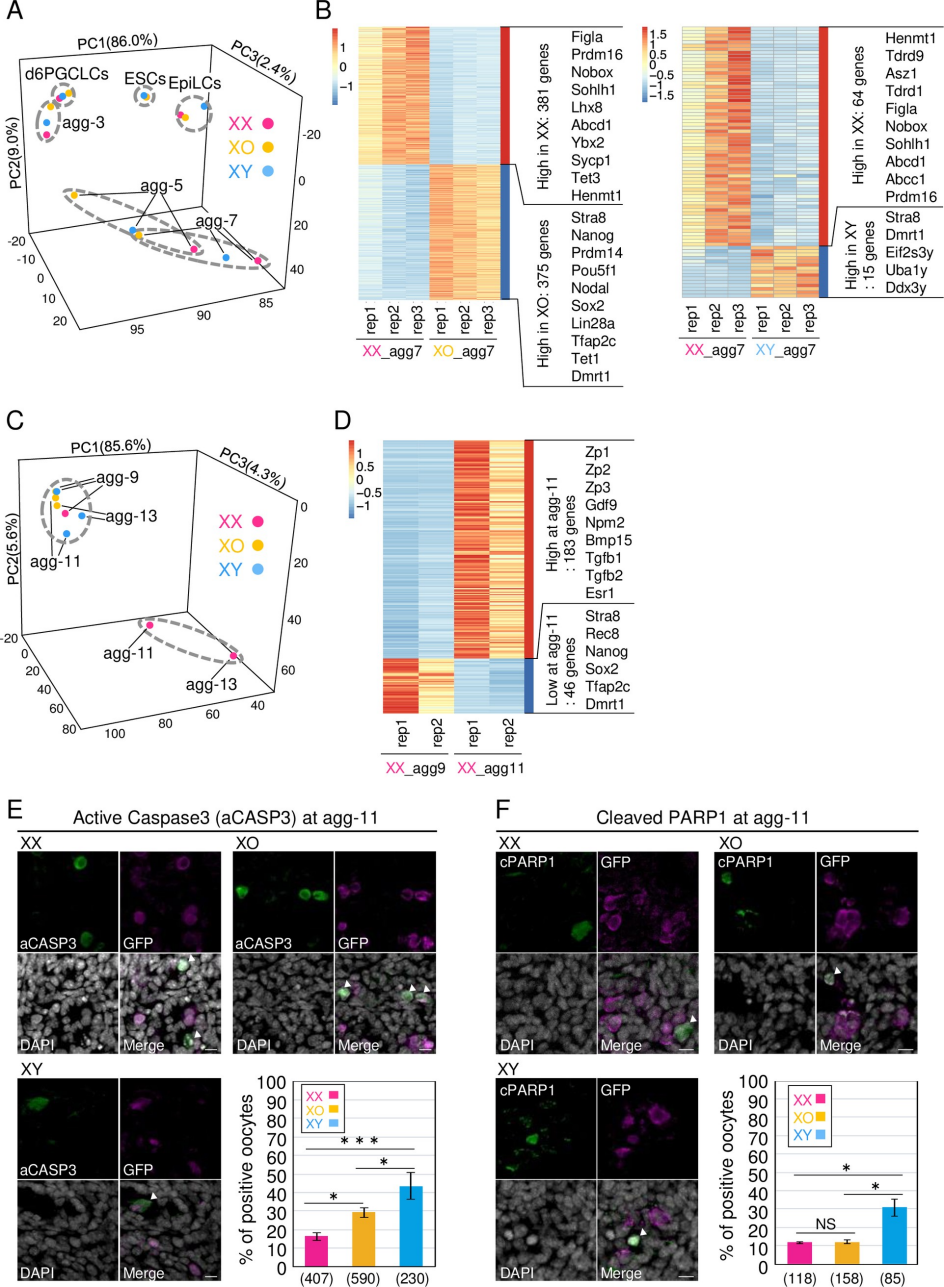
Transcriptome analysis of XX, XO and XY oogenesis *in vitro*. (**A**) PCA analysis of ESCs, EpiLCs, PGCLCs and PGCLCs/oocytes in agg-3-7. Each dot represents the data compiled from three independent experiments, except in the case of ESCs, where the dots represent the data from two independent experiments. (**B**) Heatmap analysis of the transcriptome of agg-7. The heatmap and clustering are based on the average of the transcripts per million (TPM) gene expression levels. The number of DEGs and representative genes are shown at the right of the heatmap. (**C**) PCA analysis of PGCLCs/oocytes in agg-9-13. Each dot represents the data compiled from two independent experiments. (**D**) Heatmap analysis of the transcriptomes of agg-9 and -11. The heatmap and clustering are based on the average of the transcripts per million (TPM) gene expression levels. The number of DEGs and representative genes are shown at the right of the heatmap. (**E**) Immunostaining of active Caspase 3. The graph depicts the results of the immunostaining analysis. (**F**) Immunostaining of cleaved PARP1. The graph depicts the results of the immunostaining analysis. Scale bars, 10 μm. The data was compiled from at least three independent experiments. *P* values were calculated by Tukey’s HSD test. ***P<0.001, *P<0.05; NS, not significant.

To gain insights into the reasons for the compromised differentiation of XO and XY oocytes, gene expression profiles were determined by RNA-seq analyses using ESCs, EpiLCs, PGCLCs and oocytes at 3 (agg-3), 5 (agg-5), 7 (agg-7), 9 (agg-9), 11 (agg-11) and 13 days (agg-13) of culture. Principal component analysis (PCA) of the expression profiles showed that the expression profiles of XX, XO and XY were similar before and at agg-3 (Fig 2A), suggesting that differentiation from ESCs to early oocytes underwent in a similar manner. At agg-5 and -7, dynamic alteration of gene expression profile was observed in XX oocytes, but such alteration was observed to a lesser degree in XO and XY oocytes (Fig 2A), suggesting that the differentiation of XO and XY oocytes was delayed. This was not due to a difference in the somatic cell environment, since the expression of key genes, such as RA-related genes and BMPs, was comparable among rOvaries harboring XX, XO or XY oocytes (S3 Fig). Analysis of the differentially expressed genes (DEGs) at agg-7 revealed that the genes related to an advanced stage of oogenesis, such as *Nobox*, *Figla* and *Sohlh1*, were enriched in XX oocytes (Fig 2B). In contrast, genes related to PGCs or the early meiotic process, such as *Pou5f1*, *Nanog*, *Sox2* and *Stra8*, were enriched in XO. This was also the case in XY, although to a lesser degree: *Stra8* and several Y-linked genes were enriched in XY. This transcriptome analysis indicated that meiosis entry and/or progression was delayed in XO and XY oocytes, compared to XX oocytes. It was also clear that the gene expression profile was dynamically changed between agg-9 and agg-11, and further changed at agg-13, in XX oocytes (Fig 2C). In contrast, only a subtle change of gene expression profiles was observed during the same period in XO and XY oocytes (Fig 2C). The DEGs upregulated during the period were genes involved in oocyte growth, such as *Zp1*, *Zp2*, *Zp3*, *Gdf9*, *Npm2* and *Bmp15*, and the DEGs downregulated were genes involved in meiotic prophase I or pluripotency, such as *Stra8*, *Rec8*, *Nanog* and *Sox2* (Fig 2D). This observation indicates that the differentiation of XO and XY oocytes was arrested before the initiation of oocyte growth. Consistent with this suggestion, the number of XO and XY oocytes rapidly dropped after agg-11 (Fig 1D). Moreover, two typical apoptosis markers, active Caspase 3 and cleaved PARP1, were more frequently observed in XO and/or XY oocytes than XX oocytes (Fig 2E and 2F). Collectively, these transcriptome and immunofluorescence analyses indicated that the lower productivity of XO and XY oocytes was mainly due to a delay in the early differentiation process and termination before oocyte growth.

### Delayed meiotic entry and progression in XO and XY oocytes

**Fig 3:**
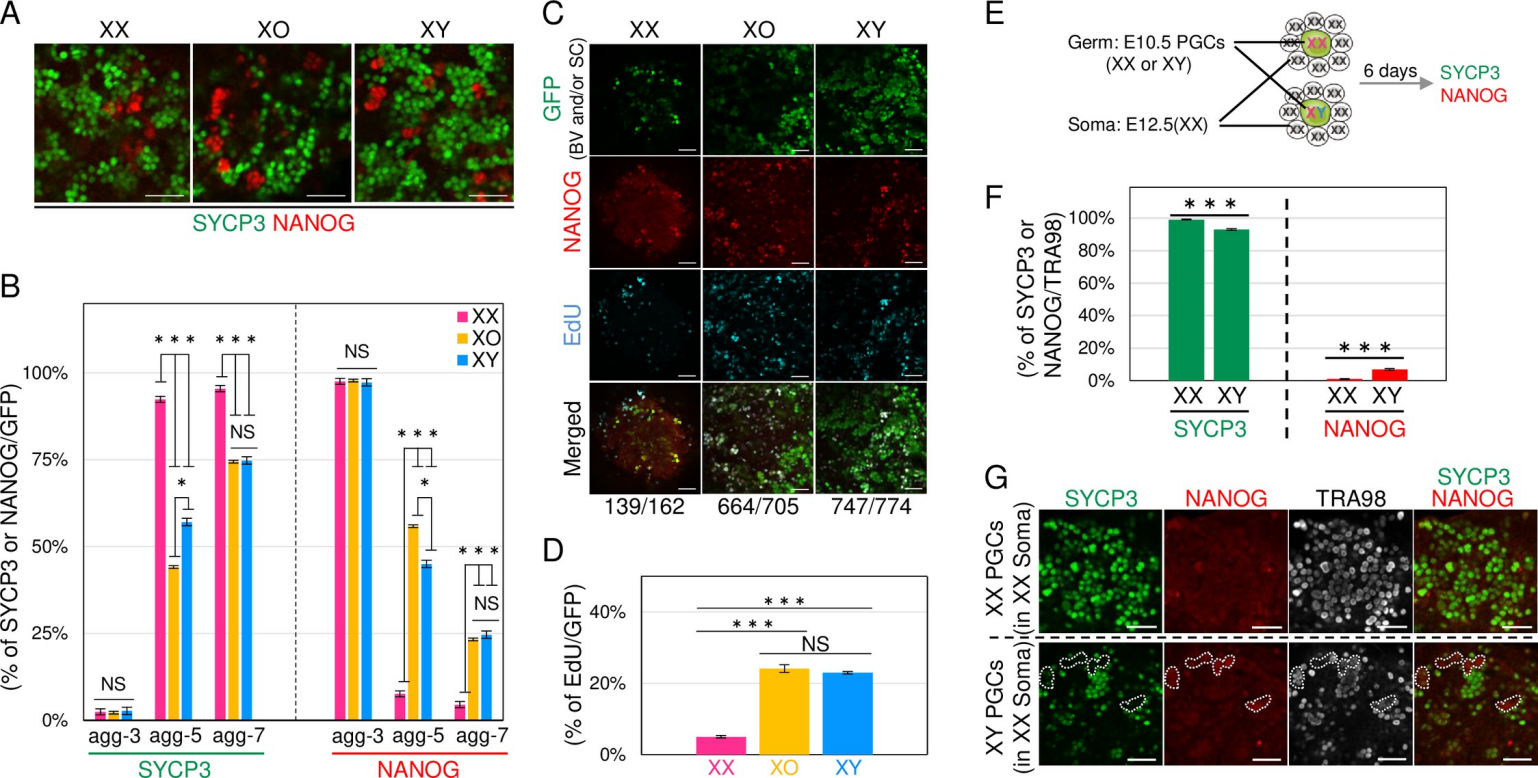
Meiotic initiation and progression in XX, XO and XY PGCLC/oocytes. (**A**) Meiotic initiation in XX, XO and XY PGCLC/oocytes. Images show the immunofluorescence analysis of SYCP3 (green) and NANOG (red) in PGCLCs/oocytes at agg-7. Scale bars, 50 μm. (**B**) Time-course of meiotic entry. The graph shows the average percentage and SD of SYCP3- or NANOG-positive cells at the days indicated. The data were compiled from two independent experiments. (**C**) EdU incorporation into NANOG-positive cells. Images show the immunofluorescence analysis of BV/SC (green), NANOG (red), EdU (Blue) and their merged images in rOvaries. The number below the images indicates NANOG-positive cells per EdU-positive cells. Scale bars, 50 μm. (**D**) The percentage of EdU-positive cells per BV- and/or SC-positive cells. The graph indicates the average percentage and SD of EdU-positive cells per BV- and/or SC-positive cells. The data were compiled from two independent experiments. (**E**) Schematic diagram of the reconstitution experiment using PGCs derived in vivo. XX or XY PGCs of E10.5 embryos were reaggregated with gonadal somatic cells of E12.5 female embryos. (**F**) The percentage of cells entering meiosis. The graph shows the average percentage and SD of SYCP3- or NANOG-positive cells at the days indicated. The data was compiled from four independent experiments. *P* values were calculated by *t*-test. ***P<0.001. (**G**) NANOG-positive XY germ cells in the rOvary. Images show the immunofluorescence analysis of SYCP3 (green), NANOG (red), TRA98 (a marker of germ cells: white) and their merged images. The dashed lines indicate representative NANOG-positive germ cells. Scale bars, 50 μm.

As described above, the transcriptome analysis indicated that the timing of the meiotic initiation was delayed in XO and XY PGCLCs/oocytes. To confirm this, the expressions of SYCP3, NANOG and GFP (for BV and SC), which are markers of meiotic entry, pluripotency and PGCs/oocytes from ESCs, respectively, were examined at various stages of IVDi culture by immunofluorescence analysis. Among XX PGCLCs/oocytes, almost all (95.5%) were positive for SYCP3, demonstrating that they entered meiosis by agg-7 (Fig 3A and 3B). In contrast, a certain population (about 25%) of XO or XY PGCLCs/oocytes remained negative for SYCP3 but positive for NANOG at agg-7 (Fig 3A and 3B). The percentage of SYCP3-negative and NANOG-positive PGCLCs was significantly higher in XO and XY than in XX at agg-5 and -7. EdU incorporation was observed in 25% of XO or XY PGCLCs/oocytes, most of which were NANOG-positive PGCLCs (Fig 3C and 3D), reinforcing the notion that NANOG-positive PGCLCs were still mitotic. This is at least partly consistent with evidence that the numbers of XY PGCLCs/oocytes at agg-9 were significantly higher than the numbers of XX oocytes (Fig 1D). These results demonstrated that the timing of meiotic initiation was indeed delayed in XO and XY PGCLCs/oocytes. However, this finding appeared to be inconsistent with a previous report in which meiotic entry was shown to be regulated by the somatic environment, irrespective of the sex of germ cells [3]. To investigate this discrepancy, we repeated the previous experiment with slight modifications using PGCs derived *in vivo* (Fig 3E). When E10.5 XX- or XY-PGCs were aggregated with E12.5 female gonadal somatic cells, a delay of meiotic initiation was still observed (Fig 3F). Although the difference between XX and XY PGCs was lower than the difference between XX and XY PGCLCs, the percentage of SYCP3-positive oocytes was significantly lower in XY than in XX, and *vice versa*: the percentage of NANOG-positive PGCs was significantly higher in XY than in XX (Fig 3F and 3G). These results were consistent with the previous report: the percentage of meiotic cells was slightly lower in XY germ cells (84%, n = 271) than in XX (93%, n = 260), although a statistical analysis was not performed in the study [3]. In addition, a similar trend was observed in the gene expression profile of *Sry*-mutant XY female (XY^**Δ**Sry^) [18]: *Nanog* expression was higher in E13.5 XY^**Δ**Sry^ PGCs than in E13.5 XX PGCs, and *Sycp3* expression was lower in in E13.5 XY^**Δ**Sry^ PGCs than in E13.5 XX PGCs (S4 Fig). Collectively, these results demonstrate that the timing of the meiotic initiation was delayed in XO and XY PGCLCs/oocytes.

### Mispairing of sex chromosome(s) in XO and XY oocytes

**Fig 4:**
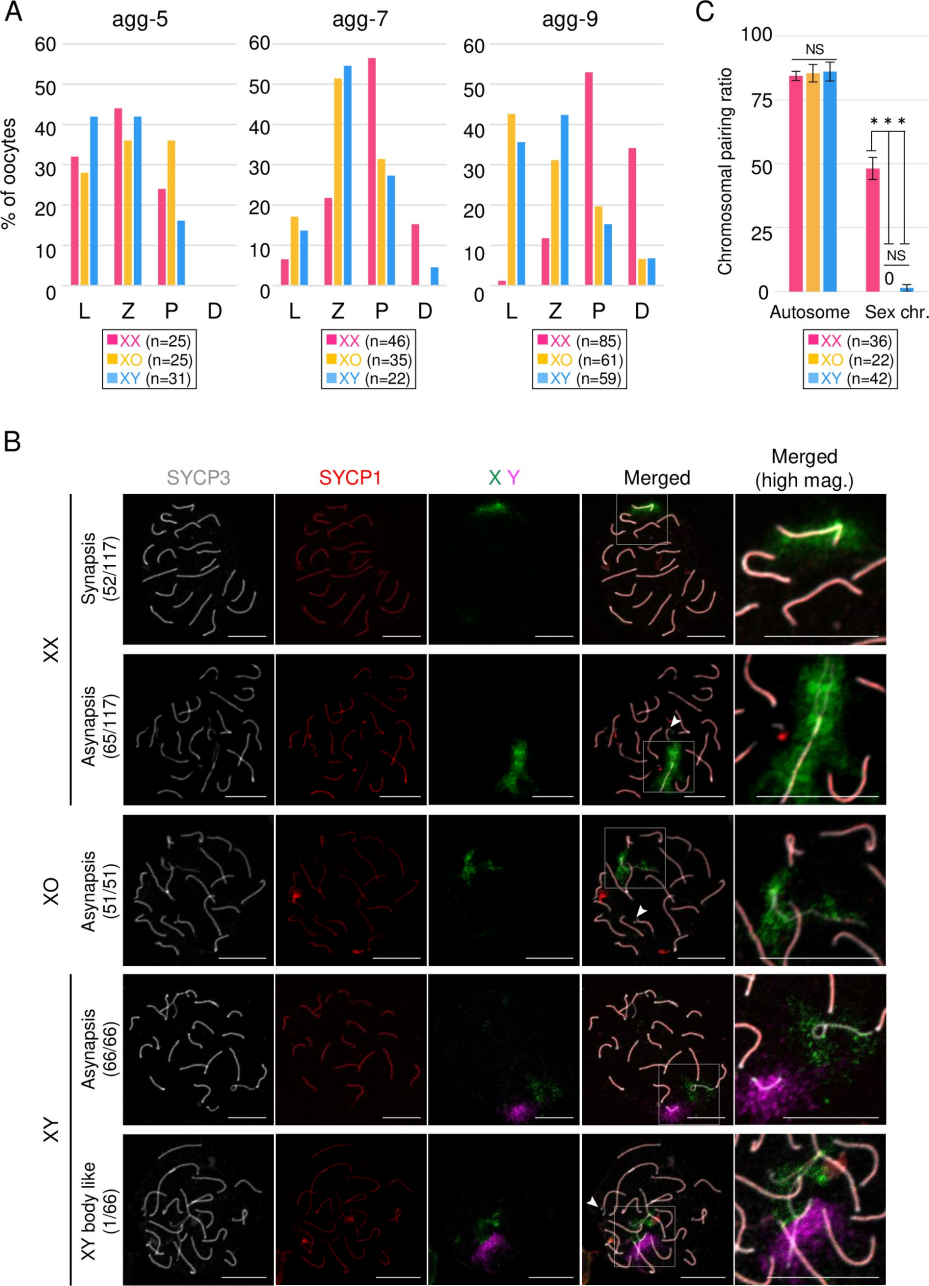
Meiotic progression and chromosome paring in XX, XO and XY oocytes. (**A**) Meiotic progression in XX, XO and XY oocytes. The graphs show the percentages of the meiotic stage at the day of culture indicated. L, leptotene; Z, zygotene; P, pachytene; D, diplotene. (**B**) Pairing of homologous chromosomes in XX, XO and XY oocytes. Images show the immunofluorescence analysis of SYCP3 (white) and SYCP1 (red), and FISH analysis of the X chromosome (green) and Y chromosome (purple). The dashed squares in the merged images are shown at high magnification (right). The numbers of samples showing the phenotype are shown with the total number tested (left). Arrowheads indicate asynapsed bivalents at the end of the chromosomes. Scale bars, 10 μm. (**C**) Pairing rates of autosomes and sex chromosomes. Each value was calculated from three independent experiments (see also Materials and Methods). *P* values were calculated by Tukey’s HSD test. ***P<0.001, **P<0.01, *P<0.05; NS, not significant.

In order to gain a deeper understanding of the delayed oogenesis in XO and XY oocytes, the progression of meiotic prophase I, in which homologous chromosomes pair through leptotene, zygotene, pachytene and diplotene stages, was determined on the basis of a previous report [19]. At agg-5, the percentage of oocytes in each stage was not clearly different among the XX, XO and XY oocytes (Fig 4A), though we should consider that a small number of endogenous oocytes (usually less than 10% of oocytes [16]) would have been in the pachytene and diplotene stages. Therefore, the pachytene oocytes at this stage may have been derived from endogenous oocytes. At agg-7, about 50% of XX oocytes proceeded to the pachytene or subsequent diplotene stage. In contrast, more than 50% of XO and XY oocytes were still at the zygotene stage. At agg-9, more than 30% of XX oocytes proceeded to the diplotene stage, whereas the percentage of XO and XY oocytes at the zygotene stage remained constant (Fig 4A). On the other hand, the percentage of XO and XY oocytes at the leptotene stage was increased. This may have been due to cells that *de novo* entered meiosis at this stage. Based on these observations, XO and XY oocytes were likely arrested at the zygotene stage. Since meiotic arrest at the zygotene stage is caused by mispairing of homologous chromosomes, we analyzed the asynapsis rate in XX, XO and XY oocytes. We counted oocytes that have more than 1 pair of homologous chromosomes that are completely aligned, based on immunofluorescence analysis of SYCP1 and SYCP3. Under this criterion, 44.4% of XX oocytes completed the synapsis of all homologous chromosomes (Fig 4B). In contrast, we could not find any XO or XY oocytes that completed synapsis. This may have been simply due to a lack of chromosomes homologous to the X chromosome in XO or XY oocytes. To evaluate this possibility, we performed fluorescence *in situ* hybridization to identify sex chromosomes. As expected, none of the X chromosomes in XO oocytes were paired (Fig 4B). Among the samples tested, we could not detect a typical self-pairing of X chromosomes. In XY oocytes, the sex chromosomes were separated, with the exception that 1 of the 66 oocytes showed paired sex chromosomes (Fig 4B). The rare pairing of the sex chromosomes in XY oocytes is consistent with a previous result showing rare formation of the XY body in the XY^TIR^ female [20, 21]. Interestingly, the pairing rate of autosomes was not different between XX, XO and XY oocytes, whereas the sex chromosomes were more prone to mispairing than autosomes even in XX oocytes (Fig 4C). These findings indicated that the sex chromosomes were distinct from autosomes in terms of the pairing of homologous chromosomes during meiotic prophase I. Because the asynapsis domain(s) of the chromosome are marked by phosphorylated H2AX (γH2AX), the cloudy signals of γH2AX were detectable at a majority of the chromosomal domains stained with SYCP3 but not SYCP1, which were not paired regions (S5A Fig). There were several partial asynapses observed at the end of the autosomes (Fig 4B, arrowheads). Therefore, we classified the pattern of asynapsed bivalents, and found that asynapsis at the end was most frequent in XX, XO and XY oocytes, and the distribution of the patterns was not different among these genotypes (S5B Fig). It is known that asynapsis of chromosome(s) activates checkpoint protein CHK2 [22]. Therefore, we tested whether inhibitors of CHK2 can rescue the loss of XO and XY oocytes. Although we used three different commercially available inhibitors (C3742, PV1019 and CCT241533) at the highest concentration that did not affect oocyte viability, none of them restored the number of XO and XY oocytes (S6A Fig). This indicates that a CHK2-independent pathway is involved in the elimination of these oocytes. Supporting this idea, the frequency of oocytes with phosphorylated CHK2 (pCHK2), an activated form of CHK2, was comparable between XX, XO and XY oocytes in rOvaries as well as P1 ovaries *in vivo* (S6B Fig). Further, such involvement of a CHK2-independent pathway might be consistent with a recently proposed model that oocytes with a smaller number of asynapses are eliminated by a CHK2-independent pathway [22].

Given that γH2AX induced transcriptional repression in the asynapsis regions, a phenomenon known as meiotic silencing of unsynapsed chromatin (MSUC) [23, 24], global repression could occur in sex chromosomes in the oocytes. To verify this possibility, we measured the amounts of transcripts on X chromosomes (X-transcripts) in XX, XO and XY oocytes using data from the RNA-seq analysis. The amounts of autosomal transcripts were not obviously different at any stage in all genotypes, whereas those of X-transcripts were dynamically changed in XX cells (S7A Fig). The X/A ratio (the value of the X-transcript divided by that of the autosomal transcript) of XX ESCs was 2 times higher than those of XO and XY ESCs and slightly decreased in EpiLCs (S7B Fig). The X/A ratio dropped in PGCLCs, gradually increased by agg-5 and dropped again in agg-13. When XX PGCLCs/oocytes were compared with single X PGCLCs/oocytes, the X/A ratio was around 1.3~1.5 (S7C Fig), though several X-linked genes showed more than 2 times higher expression in XX oocytes than in single X oocytes (S7D, S7E and S7F Fig). The dynamics of X/A were largely consistent with those in vivo [25]. In contrast, the amounts of X-transcripts and the X/A ratio in XO and XY germ cells remained constant from ESCs to agg-9, slightly increased after agg-9 and then became comparable to those in XX oocytes at agg13 (S7A and S7B Fig). Although MUSC was not obvious during differentiation of XO and XY oocytes, the increased X-transcripts and the increased X/A ratio may have been attributable to a loss of oocytes with mispaired chromosomes. Considering that transcriptional repression of the X chromosome by γH2AX was limited in oocytes, unlike in spermatocytes [26, 27], it is possible that partial repression at the asynapsis region covered by γH2AX occurs in XO and XY oocytes at agg-7 and -9.

### Involvement of Y-liked genes in XY oocyte formation

**Fig 5:**
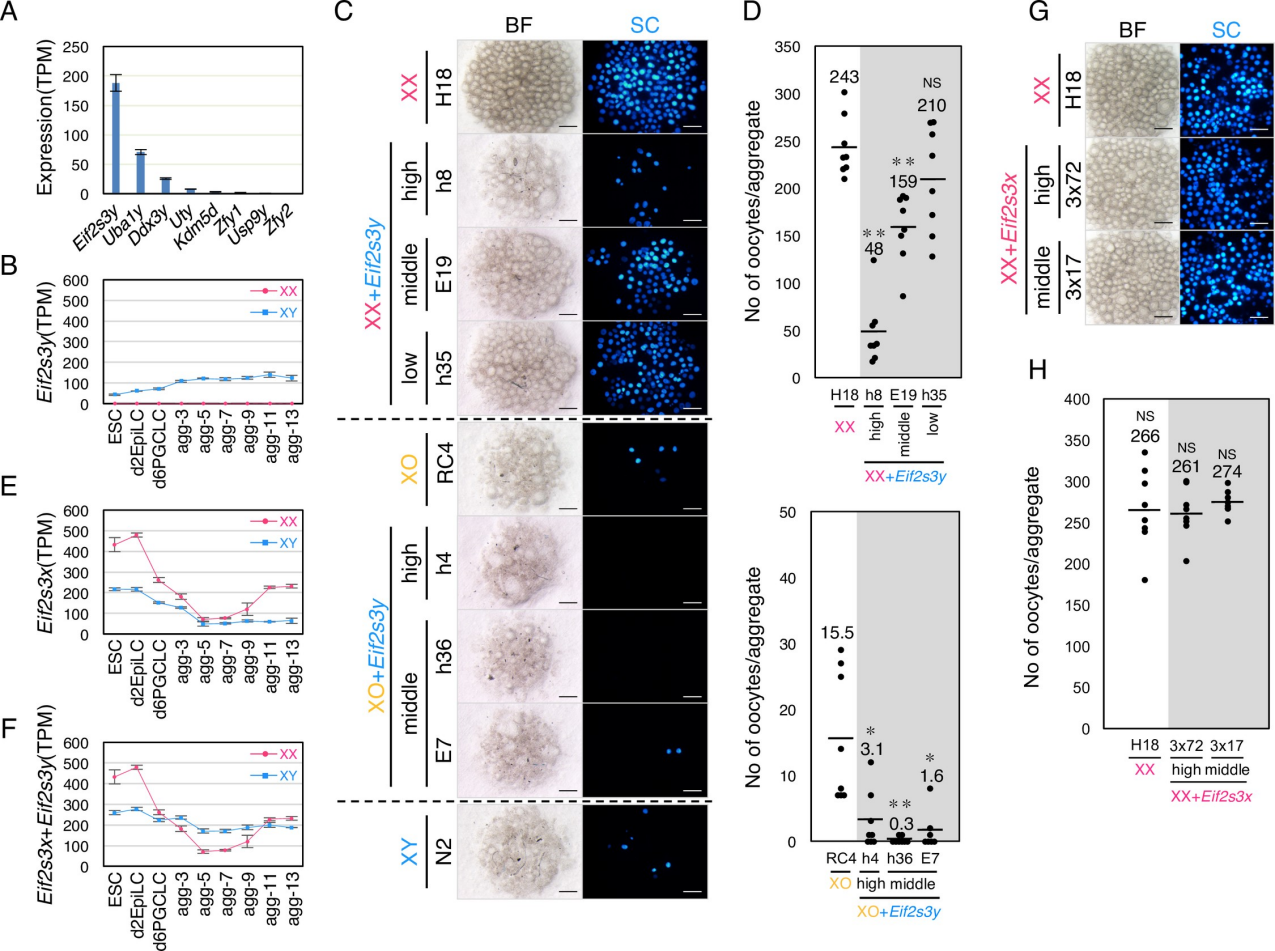
Inhibitory effect of *Eif2s3y* on oogenesis. (**A**) Expression level of Y-linked genes in XY oocytes at agg-7. Black bars indicate SD. (**B**) Expression dynamics of *Eif2s3y*. The graph shows TPM and SD of *Eif2s3y* in XX and XY cells. (**C**) Oocyte differentiation from *Eif2s3y*-expressing XX and XO ESCs. Images are rOvaries at agg-21. The expression level of *Eif2s3y* is shown as high, middle or low, based on S6B Fig. Scale bars, 200 μm. (**D**) The number of oocytes formed in culture. Each dot indicates the number of oocytes formed in one rOvary. The numbers in the graph indicate the average number of oocytes formed in each genotype. *P* values were calculated by Steel Dwass test. ***P<0.001, **P<0.01, *P<0.05; NS, not significant. (**E**) Expression dynamics of *Eif2s3x*. The graph shows TPM and SD of *Eif2s3y* in XX and XY cells. (**F**) Total amount of *Eif2s3x* and *Eif2s3y* transcripts. (**G**) Oocyte differentiation from *Eif2s3x*-expressing XX ESCs. Images are rOvaries at agg-21. The expression level of *Eif2s3x* is shown as high or middle, based on S6B Fig. Scale bars, 200 μm. (H) The number of oocytes formed in culture. Each dot indicates the number of oocytes formed in one rOvary. The numbers in the graph indicate the average number of oocytes formed in each genotype. *P* values were calculated by Steel Dwass test. ***P<0.001, **P<0.01, *P<0.05; NS, not significant.

As described above, the number of XY oocytes was lower than that of XO oocytes (Fig 1C and 1D). This suggests that Y-linked gene(s) have an inhibitory effect on early oocyte differentiation. The number of XY oocytes fell below that of XO oocytes after agg-9 (Fig 1D). A review of the gene expression profiles revealed that only 8 Y-linked genes were detectable during oogenesis in the culture system and non-growing oocytes (NGO) in XY^**Δ**Sry^ female [18] (S8 Fig). Among these genes, the transcripts of *Eif2s3y* were most abundant in XY oocytes (Figs 5A and S8). The expression of these genes was constantly observed during IVDi culture (Figs 5B and S8). Therefore, we next evaluated the inhibitory effect of *Eif2s3y* on oogenesis by overexpression of the gene in XX and XO ESCs (S9A and S9B Fig), followed by induction of oogenesis in IVDi culture. Strikingly, overexpression of *Eif2s3y* decreased the number of oocytes derived from both XX and XO ESCs (Fig 5C and 5D). The decrement was correlated to the expression level of *Eif2s3y*: ESCs with a high *Eif2s3y* expression tended to yield a lower number of oocytes. The impact of *Eif2s3y* expression was also dependent on the number of X chromosomes. XO oocytes were more influenced than XX oocytes by *Eif2s3y* expression at a similar level (Figs 5D and S9B). Whether *Eif2s3y* is the only gene responsible for the reduced number of XY oocytes was tested by induction of oocytes from *Eif2s3y*-deleted XY ESCs (S10A Fig). Although the number of SC-positive cells from the *Eif2s3y* KO ESCs at agg-11 was restored to the level of SC-positive cells from XO ESCs, the subsequent oocyte formation was hardly observed at agg-21 (S10B and S10C Fig). These findings indicate that not only *Eif2s3y* but also other Y-linked genes are involved in the reduction of XY oocytes. Since oogenesis from the *Eif2s3y* KO ESCs was partially restored at agg-11, *Eif2s3y* could play an inhibitory role on early oogenesis.

There is a homolog of *Eif2s3y* on the X chromosome, named *Eif2s3x*, which encodes an amino acid sequence that is 97.9% (461/471 amino acids) identical to EIF2S3Y. Based on the transcriptome analysis, *Eif2s3x* was expressed from ESCs to agg-13 at a different level (Fig 5E). Consistent with the dosage of X chromosomes, *Eif2s3x* expression was 2 times higher in XX ESCs than XY ESCs. The expression was repressed between agg-5 and -9, when PGCLCs entered meiosis, and then upregulated at agg-11 and -13. Since *Eif2s3y* was constantly expressed, the total amount of the *Eif2s3x* and *Eif2s3y* in XY cells was constant from ESCs to agg-13 and even higher between agg-5 and agg-9 than in XX cells (Fig 5F). To see whether *Eif2s3x* has an inhibitory effect on oogenesis similar to that of *Eif2s3y*, we induced oocytes from two *Eif2s3x*-transgenic XX ESC clones, 3x72 and 3x17, in which exogenous *Eif2s3x* was expressed at a similar level to the *Eif2s3y*-high (h8) and *Eif2s3y*-middle (E19) clones, respectively (S9A and S9C Fig). We could not obtain ESC clones that exhibited a higher expression of exogenous *Eif2s3x* than the 3x72 clone. IVD culture experiments showed that the number of oocytes from *Eif2s3x*-overexpressing clones was comparable to that of the control XX ESCs (Fig 5G and 5H). These results suggested that *Eif2s3x* and *Eif2s3y* play distinct roles on oogenesis, despite their high homology.

Genomic analysis showed that the 3’UTRs of *Eif2s3x* and *Eif2s3y* had different lengths (S11A Fig), suggesting these regions are involved in the gene expression. To evaluate the function of the 3’UTRs, each genomic fragment was inserted at the end of *mCherry* driven by the c-kit promoter (S11B Fig). ESC lines harboring each reporter construct were subjected to quantification of the fluorescence intensity of mCherry. FACS analysis revealed that the intensity was lower in ESC lines with *Eif2s3x 3’UTR*, compared to those with *Eif2s3y 3’UTR* (S11C and S11D Fig). This difference was not only due to translational repression but also mRNA degradation, since the amount of mRNA was also lower in ESC lines with *Eif2s3x 3’UTR* than those with *Eif2s3y 3’UTR* (S11E Fig). This observation was consistent with evidence that the fluorescence intensity of mCherry per mRNA was lower in ESC lines with *Eif2s3x 3’UTR* than those with *Eif2s3y 3’UTR* (S11F Fig). These findings demonstrate that not only the protein function but also the regulation of gene expression is distinct between *Eif2s3x* and *Eif2s3y*. Although the biological significance of the distinct regulation of these genes is currently unclear, it is possible that *Eif2s3x* would have a weaker inhibitory effect on oogenesis than *Eif2s3y*, and would therefore need to be repressed during the period of oogenesis. This possibility will be evaluated in the future.

## Discussion

**Fig 6:**
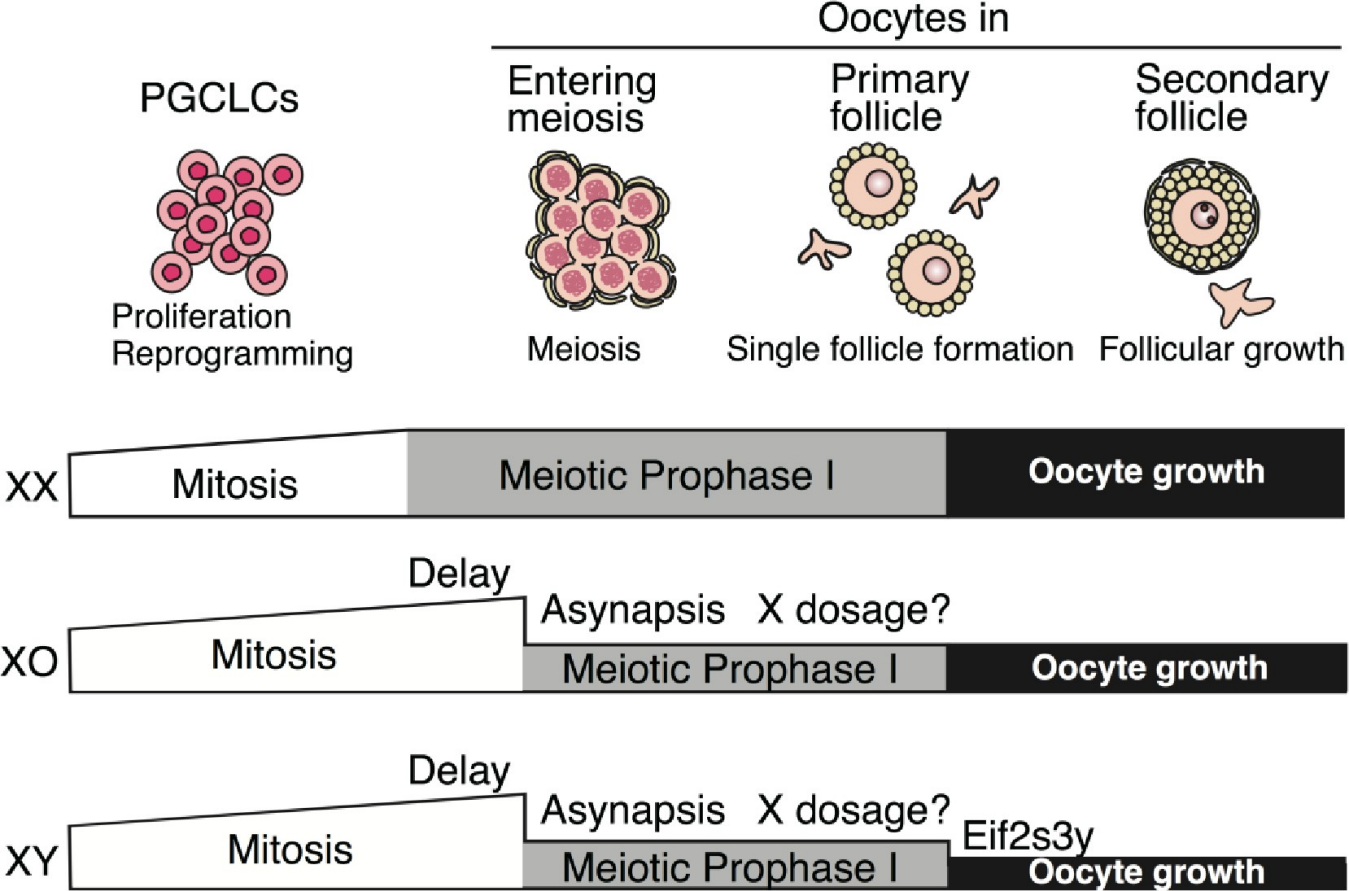
Summary of oogenesis in culture using XX, XO and XY ESCs. Differentiation of XO and XY oocytes is disrupted at three points. First, meiotic initiation was delayed in XO and XY oocytes. Second, pairing of homologous chromosomes was severely disrupted in XO and XY in oocytes. Although X-transcripts in XO and XY oocytes were lower than those in XX, how it affects to loss of oocytes is not clear. Third, *Eif3s3y* has a negative impact on oogenesis, although the molecular function remains to be clarified.

Here we analyzed oocytes derived from XX, XO and XY ESCs in order to investigate the requirement of sex chromosomes in oogenesis in culture. An advantage of the culture system was that PGCs/oocytes could be cultured under the same environmental condition. Under the condition, the numbers of oocytes from XO and XY ESCs were significantly lower than those from XX ESCs due to the delay of meiotic initiation and meiotic arrest caused by asynapsis in both XO and XY oocytes, and expression of *Eif2s3y* in XY oocytes (Fig 6). These phenomena are mostly consistent with findings from previous studies using sex-reversal animal models, reinforcing the notion that this culture system would be a useful tool to improve our understanding of the function of sex chromosomes in oogenesis. On the other hand, it was evident that the reductions in the numbers of XO and XY oocytes in culture were more severe when compared to those *in vivo*: for example, the numbers of XO and XY oocytes in neonatal ovaries were half and one-third of those observed with XX [15, 18], compared to ~one-twelfth and ~one-thirty-seventh of those observed with XX oocytes in this study. This difference may have been due to the inferior environment in culture relative to that *in vivo*, which indeed compromised meiotic pairing and developmental potency [16]. Consistent with this explanation, we observed that pairing of sex chromosomes, including self-pairing of the single X chromosome in XO or XY, which is necessary to escape from cell death (see below), was less efficient in culture than *in vivo* (Fig 4B and 4C). In the environment reconstituted *in vitro*, oocytes may be prone to cell death that is avoidable *in vivo*. For these reasons, further refinement is required to render the culture system more robust.

The delay of meiotic initiation was observed not only in XO and XY PGCLCs but also in XY PGCs from E10.5 (Fig 3A, 3B, 3E and 3F). A similar delay was reported in XY PGCs from E11.5 cultured in female gonadal somatic cells from E12.5 [3]. Such a delay was also observed in XY^TIR^ female mice, a sex-reversal strain caused by malfunction of the Y chromosome [28] and in *Sry*-mutant XY female (XY^**Δ**Sry^) [18] (S4 Fig). These results indicate that XO and XY germ cells are refractory to meiotic entry during oogenesis, compared to XX germ cells. The different timing may be attributed to the epigenetic difference between female and male PGCs. It is known that a number of meiotic genes are regulated by CpG methylation [29–31]. In PGCs, there are several genomic regions that are differentially methylated in a sex-dependent manner [32]. Moreover, the level of genome-wide CpG methylation in ESCs harboring two X chromosomes is lower than that in ESCs harboring a single X chromosome [31, 33]. Therefore, it is possible that two X chromosomes ensure the timing of meiotic entry through the regulation of CpG methylation. Although the X/A ratio of XX oocytes was 1.3~1.5 times higher than that of single X PGCLCs/oocytes, we found X-linked genes whose transcripts were more than 2 times higher in XX oocytes than in XO or XY oocytes (S7C and S7F Fig). Among these genes, *Yy2* could be an interesting candidate for involvement in the timing of meiotic entry. *Yy2* is a paralog of *Yy1* and in some cases plays roles opposite to those of *Yy1* by competing with the DNA-binding activity of *Yy1* [34–36]. Interestingly, *Yy1* is involved in regulation of X chromosome inactivation [37], suggesting a possible contribution of *Yy2* to reactivation of the X chromosome, a hallmark of XX PGC-specific epigenetic reprogramming. It could be interesting to elucidate the function of *Yy2* in the proper timing of meiotic initiation. In our experiments, it appeared that the expression profiles of XY oocytes were closer to that of XX oocytes than XO oocytes (Fig 2A). The reason for this is currently unclear, but it is possible that Y-linked gene(s) may play a positive role in early meiotic progression, and/or that Y chromosome promotes, perhaps temporally, the pairing of sex chromosomes that are required for meiotic progression.

Another possible reason for the loss of XO and XY oocytes is the mispairing of homologous chromosomes during meiotic prophase I (Fig 6) [11, 20, 23, 24]. Indeed, almost all XO and XY oocytes in the culture system showed mispairing (Fig 4B). It is thought that oocytes with mispairing of homologous chromosomes were eliminated by a meiotic checkpoint system, of which machinery in the female germ line is not as well characterized as that in males. Disruption of the genes involved in, for example, meiotic recombination arrests spermatogenesis precisely at the pachytene stage, whereas the arrest is less stage-specific in oocytes [38]. In this culture system, the number of XO and XY oocytes rapidly dropped after agg-11 (Fig 1D), suggesting that the meiotic checkpoint is evoked at this stage. RNA-seq analysis clearly revealed that the transcriptomes of XO and XY oocytes at agg-11 and agg-13 were arrested at this stage, whereas the transcriptome of XX oocytes at agg-11 and agg-13 proceeded beyond the pachytene stage (Fig 2D). DEGs between agg-9 and agg-11 illustrated that oocytes switched from meiotic prophase I to oocyte growth during this stage. Consistent with this idea, we found that active Caspase 3 and/or cleaved PARP1 was more frequently observed in XO and XY oocytes than in XX oocytes at agg-11 (Fig 2E and 2F), suggesting that the oocyte elimination is mediated by apoptosis. In contrast, the number of oocytes positive for pCHK was comparable between XX, XO and XY oocytes at agg-11 (S6B Fig). As proposed previously, there are two distinct types of pathways, a CHK2-dependent and a CHK2-independent pathways, to eliminate aberrant oocytes having mispaired chromosome(s) and/or DSB [22, 39]. Based on these studies, oocytes with a small number of asynapses would be eliminated by a CHK2-independent pathway. Our results showed that almost all oocytes (51 of 51 XO oocytes and 65 of 66 XY oocytes) fail in pairing or self-pairing of the sex chromosome(s), whereas the pairing rate of the autosomes was comparable among the XX, XO and XY oocytes (Fig 4B and 4C). This suggests that XO and XY oocytes are eliminated by a CHK2-independent pathway. A major outcome of the CHK2-independent pathway to eliminate oocytes is MUSC [22–24]. However, we did not observe obvious MUSC in the transcriptome analysis. This may have been due to delayed meiotic initiation and loss of XO and XY oocytes with MUSC in the culture system.

Our results suggested that the reason the extent of oocyte reduction *in vivo* differed from that *in vitro* was that the culture system was less efficient for the pairing of homologous chromosomes in meiotic prophase I than the endogenous condition *in vivo*. Specifically, it was shown that there was tendency for mispairing of X chromosome(s) not only in XO and XY but also in XX oocytes in culture (Fig 4B). It is noteworthy that even in XX oocytes, the X chromosomes were more prone to mispairing (Fig 4C) compared to the autosomes. The distinct mechanism controlling the pairing of X chromosomes in the oocyte remains unclear. Considering that meiosis-specific non-coding RNA is involved in chromosomal pairing [40], the mechanism may be related to the transcriptional activity of X chromosomes. It is known that the transcriptional activity of X chromosomes is dynamically changed during PGC differentiation: one of the X chromosomes in females is inactivated in nascent PGCs and becomes reactivated in developing PGCs [41, 42]. A recent report indicates that the transcriptional activities of two X chromosomes are equivalent at the pachytene stage [25]. Based on these findings, it is possible that completion of X chromosome reactivation, which equalizes the transcriptional activity of two X chromosomes, is prerequisite to the pairing of X chromosomes. Although the dynamics of X-transcript in culture was largely consistent with that *in vivo*, the level was slightly lower in culture. It would be worth verifying the correlation between transcriptional activity and the pairing of X chromosomes at the single cell level. In addition, we could not detect a typical self-pairing of the X chromosome in XO oocytes *in vitro*, whereas previous reports described such a structure *in vivo* [11, 23, 24]. This could also be a possible reason for the reduced number of XO oocytes *in vitro*, as a self-paired X chromosome could have escaped from transcriptional repression and presumably the meiotic checkpoint. The difference in behavior of the X chromosome could be attributed to a suboptimal condition in culture.

XY females produce fewer oocytes and offspring than XO females [11, 15], which indicates that the Y chromosome has a negative impact on oocyte production. The inferior fertility of XY females compared to XO females was attributed to both the reduction in the oocyte pool in the ovaries and the impairment of oocyte maturation and embryonic development. A genetic analysis identified *Zfy2* as a Y-linked gene that is partly responsible for the inferior fertility in XY females [15]. In XO females harboring the *Zfy2* transgene, meiotic division and subsequent polar body extrusion were impaired in the oocytes, resulting in developmental arrest of embryos at the 2-cell stage. However, the Y-linked gene(s) responsible for the reduction in the oocyte pool of XY females has remained unclear. Here we showed that *Eif2s3y* is a novel inhibitory factor of early oogenesis (Fig 6). The inhibitory effect of *Eif2s3y* overexpression was greater in XO than XX. Our results showed that the expression level of *Eif2s3y* is critical for the effect in XX oocytes: it had a negative correlation to the number of oocytes formed in culture (Fig 5C and 5D). *Eif2s3y* plays an essential role in the proliferation of spermatogonia: disruption of *Eif2s3y* causes male infertility due to the retardation of early spermatogenesis [43]. Interestingly, expression of this gene is sufficient for producing functional round spermatids in XO mice carrying *Sry* [44], indicating that *Eif2s3y* plays opposite roles in male and female gametogenesis. The molecular mechanisms of this conflictive function are currently unknown. Since *Eif2s3y* encodes subunits of the translation initiation factor, this possible conflict would be mediated by translational regulation, an intriguing prospect that merits future investigation.

These conflicting functions led us to focus on *Eif2s3x*, an X-linked gene which is a homolog almost identical to *Eif2s3y*. This gene is known to play a role in spermatogenesis similar to that of *Eif2s3y*, since overexpression of *Eif2s3x* and *Sox9* are also sufficient for producing functional round spermatids in XO mice [45]. In addition, *Eif2s3x* is an escapee of X inactivation [46]. These findings raising a possibility that this gene might disturb oogenesis even in XX oocytes. However, we found that *Eif2s3x* expression was repressed in oocytes between agg-5 and agg-9 (Fig 5E). The repression is at least partially mediated by mRNA degradation and translational repression (S11 Fig). More importantly, overexpression of *Eif2s3x* had no effect on oogenesis (Fig 5G and 5H). These results indicate that EIF2S3X and EIF2S3Y play substantially different roles in oogenesis, or perhaps that the inhibitory activity of EIF2S3X is much weaker. The latter possibility is consistent with the previous reports that overexpression of *Eif2s3x* was less effective at promoting the production of round spermatid in XO mice carrying the *Sry* gene (XO^sry^) [44, 45]. These reports also showed a relevant result that the productivity of round spermatid in XO^sry^ was positively correlated with the expression level of exogenous *Eif2s3x*, indicating that a higher expression of *Eif2s3x* than the 3x72 clone (XX ESCs carrying a high copy number of exogenous *Eif2s3x* in this study) would show an inhibitory effect on oogenesis.

Here, we evaluated oocyte formation from ESCs harboring various sets of sex chromosomes in a novel culture system. The advantage of this culture system is that it recombines germ cells and somatic cells from independent sources, thus allowing us to evaluate germ cell-intrinsic effects on oogenesis. Experiments using animal models are less efficient than those using culture systems, especially for the evaluation of gene function through the overexpression or targeted deletion of a gene. Our culture system facilitates the functional analysis of genes, and here it was indeed revealed that *Eif2s3y* has a negative role in oogenesis. Further analyses are expected to reveal the detailed function of various genes. This culture system will also be a useful tool for the screening of small molecules that restore oogenesis in XO and XY cells/animals. Such a screening could provide a strategy to overcome infertility due to disorders involving the sex chromosomes.

## Materials and methods

### Ethics statement

All animal experiments were performed under the ethical guidelines of Kyushu University for animal use (#A28-109-4) and recombinant DNA experiments (#26–74).

### Animals

*129X1/svj*, *C57Bl/6J*, *BDF1* and *ICR* mice were purchased from Japan SLC. E10.5 PGCs and E12.5 gonadal somatic cells were collected from *ICR* embryos. E11.5 BVSC PGCs were collected from embryos obtained by mating *BDF1* female mice with *C57Bl/6J* male mice harboring BVSC.

### ESCs

BVSC reporter ESCs were established as described previously [16]. BVSC H18 and BVSC N6 are XX cell lines. BVSC N2 and BVSC N3 are XY cell lines. BVSC H18 #28C9 is an XO cell line harboring a maternal X chromosome. BVSC H18 #28C12, #39RC2, #39RC3 and #39RC4 are XO cell lines harboring a paternal X chromosome. BVSC H18 h8, E19 and h35 are over expressed *Eif2s3y* XX cell lines. BVSC H18 #39RC4 h4, h36 and E7 are over expressed *Eif2s3y* XO cell lines. These ESCs were maintained under a 2i+LIF N2B27 medium condition without feeder cells.

### Generation of XO-ESCs

XO ESCs from XX ESCs were generated according to a previous report [17]. Briefly, for targeted deletion of one X chromosome, we introduced an inverted loxP sequence into a X chromosome known as a sex chromosome elimination cassette (SCEC). The targeting vector pTK-SCEC-HS1-XSL1 contains a sex chromosome elimination cassette (SCEC) flanked by a 2.0-kb short arm and a 4.0-kb long arm. SCEC includes the DsRed reporter gene and hygromycin-resistant gene between the inverted loxP sequence. The guide RNA (gRNA) was constructed with the following oligonucleotides: for CRISPR X1-F, 5'-cac cGA GAA AAT GTC CTA TCG CCG G-3’; for CRISPR X1-R, 3'- CTC TTT TAC AGG ATA GCG GCC caa a-5’. The lowercase letters indicate nucleotides added for incorporation into the pX330 vector. The target site of Cas9 is 115 bp and 112 bp away from the end of the short arm and the long arm, respectively. The targeting vector with the pX330 expressing the gRNA was transfected into BVSC H18. The targeted integration was confirmed by southern blot using genomic DNA of the ESCs after digestion with EcoRV. To eliminate the X chromosome with SCEC, Cre-GFP was transiently expressed, and then DsRed-negative ESCs were collected using a FACS Aria II (BD Bioscience). The sorted ESCs were sub-cloned on the culture plate. Each clone was used as XO-ESCs after checking the karyotype. The gRNA sequences used in this study are listed in S1 Table.

### Southern blotting

15 μg genomic DNA of H18, #28, #30 and #39 was loaded onto 0.8% TBE agarose gel after EcoRV enzyme reaction. The DNA was then transferred to a membrane (Amersham Hybond-N, GE Healthcare Life Sciences, RPN303N) and the membrane was hybridized with DIG-labeled probe DNA (15ng/ml) overnight at 42°C. After washing the membrane, we performed chemiluminescent detection with CDP-Star Substrate (Thermo Fisher Scientific T2305). Samples were analyzed by a chemiluminescence Imaging system (Fusion Solo).

### PCR analysis to identify sex chromosomes and the origin of the X chromosome

To identify the sex chromosomes, PCR amplification was performed using the following primers: for Sexing-F, TGG ATG GTG TGG CCA ATG CYC T; for Sexing-R, CCA CCT GCA CGT TGC CCT TKG TGC CCA G). The expected sizes of the PCR fragments were 253 bp for the X chromosome and 355 bp/399 bp for the Y chromosome. To identify the origin of the X chromosome, the sequence of the locus (S1E Fig) was amplified by PCR using the following primers: for X-origin-F, ACT TGG GGT TGT TGG CCT CT; for X-origin-R, CAA GCT GGA CCA GGA AGG AGC. The PCR product was digested by SfaNI and then subjected to gel-electrophoresis. The expected sizes of the PCR fragments were 1064 bp for the maternal X chromosome and 845 bp/119 bp for the paternal X chromosome. The primer sequences used in this study are listed in S1 Table.

### Karyotyping

EpiLCs were cultured with Demecolcine solution 20 ng/ml (Sigma D1925) for 3 h. After the Demecolcine treatment, EpiLCs were harvested, resuspended in 0.075M KCl and then incubated at 37°C for 15 min. Ice-cold Carnoy's solution was added to the cell suspension and then the cells were collected by centrifugation at 1,500 rpm for 7 min. After removal of the supernatant, the cell pellet was resuspended in ice-cold Carnoy's solution. After repeating the suspension of cells with Carnoy's solution three times, the cell suspension was dropped on the slides. The slides were dried and then incubated in PBS containing DAPI for 15 min at room temperature. The numbers of chromosomes were measured under a fluorescence microscope (OLYMPUS IX73).

### Gene expression analysis

For RNA-seq analysis, cDNA libraries were constructed as described previously [16]. Briefly, poly(A)+ RNAs were purified from 200–40,000 cells or oocytes (ESCs, EpiLCs and d6PGCLCs: 10,000; IVDi agg3-13: 200–40,000), using a Dynabeads mRNA DIRECT Micro Kit (Invitrogen). SC-positive oocytes were collected using a FACS Aria II (BD Bioscience). Biologically duplicated (ESCs, EpiLCs, agg-9, agg-11 and agg-13) or triplicated (PGCLCs, agg-3, agg-5 and agg-7) were prepared. Purified RNAs were subjected to library construction using a NEBNext Ultra non-Directional RNA Library Prep Kit for Illumina (NEB) for ESCs, EpiLCs, PGCLCs and agg3-7 and a NEBNext Ultra II Directional RNA Library Prep Kit for Illumina (NEB) for agg9-13. cDNAs were enriched by 12-cycle PCR. Sequencing of the libraries was performed with a HiSeq 2500 (Illumina). Obtained sequence reads were processed with the FASTX tool kit [47] to remove short (<20 bp) and low quality (quality score <20) reads, followed by trimming of the adaptor sequence. Processed reads were mapped to the mouse mm10 genome using STAR [48]. For the counting of uniquely and multiply mapped reads on RefSeq mRNAs, featureCount [49] was used with parameter -O -s 0 or -M -O -s 0, respectively. Since we noticed that some of the libraries showed abnormally high levels of Interleukin 2 (*Il2*) transcript presumably because of contamination, we therefore excluded Il2 for further analysis. For the differential gene expression analyses, edgeR [50] was used. *Eif2s3x* and *Eif2s3y* expression levels were determined with multi-mapped reads. Hierarchical clustering and principal component analyses were performed with R, based on RefSeq gene expression levels.

For quantitative PCR (Q-PCR) analysis, total RNAs were purified using an RNeasy Micro Kit (QIAGEN, 74004) and reverse-transcribed by PrimeScript 1^st^ strand cDNA Synthesis Kit (Takara, 6110A). Genes of interest were amplified with Power SYBR Green PCR Master Mix (Applied Biosystems, 4368708) on a real-time qPCR system (Biorad, CFX384).

### Immunofluorescence analysis

For H3K27me3, 2,000 EpiLCs were aggregated on a low binding U-bottom 96-well plate (NUNC) for 2 days. The aggregates were dissociated and spread on the slide glass by Cytospin (Thermo). Slides were fixed with 4% paraformaldehyde (PFA) for 30 min, washed three times in PBSTB (PBS containing 0.1% Triton X-100 and 0.1% BSA), and then incubated in anti-H3K27me3 rabbit polyclonal antibody (pAb) (Upstate 07–449) overnight at 4°C. The slides were then washed three times and incubated with anti-rabbit IgG goat pAb Alexa 488 (Invitrogen A11034) and DAPI overnight at 4°C. The slides were washed three times and covered with Fluoro-KEEPER antifade reagent (Nakalai) for analysis by a confocal microscope (Zeiss LSM 700).

For whole-mount immunofluorescence analysis, rOvaries were fixed with 4% PFA, washed twice in PBS containing 3% BSA, permeabilized in PBS containing 0.5% Triton X-100, incubated in blocking reagent (PBS containing 0.1% BSA and 0.3% Triton X-100) and then incubated with anti-GFP chicken pAb (Abcam ab13970) or anti-TRA98 rat IgG monoclonal antibody (mAb) (Bioacademia 73–003), anti-NANOG rabbit mAb (CST #8822) and anti-SYCP3 mouse mAb (Abcam ab97672) overnight at 4°C. rOvaries were then washed twice for 15 min each and three times for 1 h each and incubated with anti-chicken IgY goat pAb Alexa 488 (Invitrogen A11039) or anti-rat IgG donkey pAb Alexa 488 (Invitrogen A21208), anti-rabbit IgG donkey pAb Alexa 568 (Invitrogen A10042), anti-mouse IgG donkey pAb Alexa 647 (Invitrogen A31571) and DAPI overnight at 4°C. Finally, rOvaries were washed twice in PBS containing 0.3% Triton X-100 and soaked in Fluoro-KEEPER antifade reagent (Nakalai) for analysis by a confocal microscope (Zeiss LSM 700).

For frozen sections, freshly isolated P1 ovaries and rOvaries were fixed with 4% PFA for 1 hour at 4°C. After washing with PBS, fixed samples were sequentially soaked in 10%, 15%, and 20% sucrose and then treated with fresh 20% sucrose overnight. Then, the samples were embedded in optimal cutting temperature (O.C.T.) compound (Tissue-Tek) and sectioned at 7 μm thickness. Sections were incubated in blocking reagent (PBS containing 3% FBS and 0.1% TritonX-100) for 1 hour at room temperature. Then, primary antibodies for GFP or TRA98 and pCHK2 or active Caspase3 or cleaved PARP1 were reacted at 4°C overnight. The sections were washed three times in PBS containing 0.1% TritonX-100 and once in PBS, and then anti-chicken IgY goat pAb Alexa 488 (Invitrogen A11039) or anti-rat IgG donkey pAb Alexa 488 (Invitrogen A21208), anti-rabbit IgG goat pAb Alexa plus 555 (Invitrogen A32732) or anti-rabbit IgG donkey pAb Alexa 568 (Invitrogen A10042) and DAPI (1 μg/ml) were reacted for 1 hour at room temperature. The slides were washed three times in PBS containing 0.1% TritonX-100 and once in PBS and then mounted in Fluoro-KEEPER antifade reagent (Nakalai) for analysis by a confocal microscope (Zeiss LSM 900). The antibodies used in this study are listed in S2 Table.

### EdU analysis

A Click-iT Plus EdU Alexa Flour 594 imaging kit (Thermo Fisher Scientific C10639) was used for EdU analysis. rOvaries were cultured in 5 μM EdU labeling solution for 4 h at 37°C. The following processes were the same as for the whole-mount immunofluorescence analysis of rOvaries except for the secondary antibody reaction. rOvaries were incubated with anti-chicken IgY goat pAb Alexa 488 (Invitrogen A11039), anti-rabbit IgG donkey pAb Alexa 647 (Invitrogen A31573) and DAPI. rOvaries were soaked in Fluoro-KEEPER antifade reagent (Nakalai) for analysis by a confocal microscope (Zeiss LSM 700).

### rOvaries from E10.5 PGCs and E12.5 gonadal somatic cells

E10.5 embryos were collected from pregnant *ICR* female mice mated with *ICR* male mice. Genomic DNAs were extracted from the heads of the embryos. XX and XY were discriminated by PCR. After genotyping, female genital ridges were isolated from the embryos, dissociated by trypsin treatment, and then incubated with FITC-SSEA1 (Biolegend, #125611) for 15 min on ice. SSEA1-positive PGCs were sorted using a FACS Aria II (BD Bioscience). Each rOvary was composed of 2,000 PGCs and 30,000 gonadal somatic cells. After 6 days of IVDi culture, the rOvaries were analyzed by immunofluorescence analysis.

### Cell spreads for meiotic chromosome analysis

Chromosome spreads were processed for immunohistochemistry as previously described [16]. Briefly, rOvaries were dissociated by incubation in CTK (0.1 mg/ml collagenase IV, 0.25% Trypsin, 20% KSR and 1 mM CaCl_2_ in PBS) for 30 min at 37°C, followed by Accutase (Nakalai) for 5 min at 37°C. Dissociated single cells were suspended in hypotonic buffer and placed on glass slides. Slides were washed three times in PBS, incubated in blocking buffer (PBS containing 10% FBS) and incubated with anti-SYCP1 rabbit pAb (Novus Biologicals NB300-229) and anti-SYCP3 mouse mAb (Abcam ab97672) overnight at 4°C. The slides were then incubated with anti-rabbit IgG goat pAb Alexa 568 (Invitrogen A11036) and anti-mouse IgG donkey pAb Alexa 647 (Invitrogen A31571). Then the slides were washed three times in PBS containing 0.05% Tween 20 and incubated with anti-γH2AXSer139 mouse mAb directly conjugated with FITC (Millipore 16-202A) and DAPI for 1 h at room temperature. Slides were washed three times in PBS containing 0.05% Tween 20 and mounted in Fluoro- KEEPER antifade reagent (Nakalai) for analysis by a confocal microscope (Zeiss LSM 700).

### Immunofluorescence and DNA FISH analysis

Chromosome spreads and immunostaining were performed as described above. The primary antibody used the following anti-SYCP1 rabbit pAb (Novus Biologicals NB300-229) and anti-SYCP3 mouse mAb (Abcam ab97672). The secondary antibody used the following anti-rabbit IgG goat pAb Alexa 405 (Invitrogen A31556) and anti-mouse IgG donkey pAb Alexa 647 (Invitrogen A31571). After chromosome spreads and immunostaining, slides were washed two times in PBS containing 0.05% Tween 20, rinsed in PBS and then fixed in 4% PFA for 10 min at room temperature. The slides were then washed three times in PBS and dehydrated by a serial incubation in ethanol: 70% for 5 min, 70%, 80%, 90% and 100% for 1 min each, and 100% for 5 min. After dehydration, the slides were incubated with probes of sex chromosomes (MetaSystems D-1420-050-FI and D-1421-050-OR) and mouse Cot-1 DNA (Invitrogen 18440016) for 5 min at 75°C for denaturing, followed by 16 to 20 h at 37°C for hybridization. Slides were washed once in 2×SSC for 5 min at room temperature and then two times in 50% formamide-containing 2×SSC for 5 min at 42°C. After cooling down, slides were soaked two times in cold 70% ethanol for 5 min and two times in cold 100% ethanol for 5 min. After dehydration, the samples on the slides were mounted with Fluoro- KEEPER antifade reagent (Nakalai) for analysis by a confocal microscope (Zeiss LSM 700).

### DNA FISH analysis

200,000 ESCs and 200,000 d6PGCLCs were transferred onto a poly-L-lysine (Sigma, P8920) coated glass coverslip in a drop of PBS. Coverslips were fixed for 10 min in 4% PFA, then permeabilized on ice for 3 min 30 sec in 0.5% TritonX-100/PBS. After dehydrating through an ethanol series, they were denatured in 70% formamide-containing 2×SSC for 30 min at 80°C and dehydrated again through an ice-cold ethanol series. They were then hybridized with fluorescent BAC probes (RP24-157H12) for *Huwe1* at 37°C overnight. The coverslips were washed three times in 50% formamide-containing 2×SSC for 7 min at 42.5°C, washed twice in 2×SSC for 5 min at 42.5°C, and then stained with DAPI (1 μg/ml) for 20 min at room temperature. The coverslips and slides were mounted in Fluoro-KEEPER antifade reagent (Nakalai) for analysis by a confocal microscope (Zeiss LSM 900).

### Determination of stage of meiotic prophase I

Stages of meiotic prophase l were determined by immunostaining of SYCP3 and SYCP1 based on the method in a previous report[19]. Leptotene was defined by fragmented SYCP3 and absence of SYCP1. Zygotene was defined by extensive SYCP3 and partial merge with SYCP1. Pachytene was defined by complete merge of SYCP3 and SYCP1 on at least 19 chromosomes. Pachytene with mispaired chromosomes was distinguished from zygotene by the presence of at least one completely paired chromosome and one partially paired chromosome. Diplotene was defined by bold and continuous SYCP3-staining and enrichment of SYCP3 at the termini of the chromosome.

### For pairing analysis

Based on immunofluorescence of SYCP1 and SYCP3, oocytes with more than 10 completely paired homologous chromosomes were subjected to pairing analysis. Autosomes and sex chromosomes were distinguished by FISH. The numbers of completely paired autosome(s) and sex chromosomes were divided by 19 and 1, respectively, to determine the pairing rate. In the case of X and Y chromosomes, chromosomes showing partial pairing were counted as paired chromosomes. The average of the pairing ratio in cells analyzed in each experiment was calculated. The experiments were performed independently three or four times and statistically processed by Tukey’s HSD test. The total numbers of oocytes counted were 36, 22, and 42 in XX, XO and XY, respectively.

## Supporting information

S1 Fig Generation of XO ESCs by target disruption of the X chromosome. (**A**) Targeted integration of an elimination cassette to the X chromosome. The elimination cassette containing the DsRed gene floxed with inverted loxP sequences was introduced into the X chromosome locus. The probes and restriction enzymes for Southern blot analysis are shown. (**B**) Southern blot analyses using the probe described in (A). Note that the vector was properly integrated in the locus in clone #28 and #39. M, size marker. (**C**) Schematic diagram of elimination of the X chromosome via Cre-loxP recombination and FACS analysis. The elimination process can be monitored by expression of DsRed. FACS analysis shows DsRed expression at the elimination process. (**D**) Detection of the inactive X chromosome. Images show the immunofluorescence analysis of H3K27me3 (red) in EpiLCs after 1 day of aggregation culture. Arrowheads indicate punctuate staining of H3K27me3 representing the inactive X chromosome. (**E**) PCR analysis to distinguish the parental X chromosome. There is a polymorphism that is sensitive to SfaNI in the genome of C57BL/6J. The region can be amplified by the primers (arrows). The image below the diagram is a gel-electrophoresis of the PCR product after digestion of the enzyme. Scale bars, 10 μm. (**F**) Karyotype of the ESC clones. The X-axis indicates the number of chromosomes. The number of nuclei counted is shown in each graph. (**G**) Loss of the X chromosome in PGCLC induction. Representative images of DNA-FISH analysis of PGCLCs at day 6 of induction from BVSC H18 ESCs (left) and the quantification of the analysis (right) are shown. Scale bars, 1 μm.
(TIF)

S2 Fig Oocyte formation from XX and XY PGCs in culture. (**A**) Oocyte differentiation from XX and XY PGCs of E11.5 embryos. The PGCs were reaggregated with gonadal somatic cells of E12.5 female embryos. Note that the SC transgene was present in E11.5 PGCs, but not in E12.5 embryos. Scale bars, 200 μm. (**B**) The number of oocytes formed in culture. Each dot indicates the number of oocytes formed in one rOvary. The numbers in the graph indicate the average number of oocytes formed in each genotype. *P* values were calculated by *t*-test. ***P<0.001.
(TIF)

S3 Fig Comparable expression of key genes in the somatic cells of rOvaries harboring XX, XO and XY oocytes. The graph shows the expression of genes related to BMP and RA signaling in the somatic cells. Somatic cells were purified from rOvaries harboring XX, XO or XY oocytes. The Y-axis shows the ΔCt value of each gene relative to the average for *Rplp0* and *Ppia*. The primer sequences used in this study are listed in S1 Table.
(TIF)

S4 Fig *Nanog*, *Stra8* and *Sycp3* expression in the E13.5 XYΔSry PGCs in vivo. The graph shows the expression of *Nanog*, *Stra8* and *Sycp3* in XX and XY^**Δ**Sry^ PGCs at E13.5. The expression profile was obtained from Sakashita et al. [18]
(TIF)

S5 Fig Mispaired chromosome and γH2AX accumulation in XX, XO and XY oocytes. (**A**) Accumulation of γH2AX in the mispaired region. Three representative immunofluorescent images of SYCP3 (green), SYCP1(red), and γH2AX (white) and their merged images in XX, XO and XY oocytes are shown. The box in the merged image is shown on the right image. Note that the asynapsis regions, which are stained by SYCP3 but not SYCP1, are covered by γH2AX. (**B**) Pattern of autosomal asynapsis. The graph shows the percentage of each asynapsis pattern. Drawings at the right side of the graph illustrate a typical form of the chromosome in each asynapsis pattern.
(TIF)

S6 Fig Oocyte elimination by a CHK2-independent mechanism. (**A**) Oocyte differentiation with CHK2-inhibitors. rOvaries harboring XX, XO or XY oocytes were cultured with the CHK2-inhibitors indicated at the left. Representative images at the day of culture indicated at the top are shown. Scale bars, 200 μm. (**B**) Immunostaining of phosphorylated CHK2 (pCHK2). Representative images of immunofluorescence analysis of pCHK2 in the P1 ovary and rOvaries harboring XX, XO or XY oocytes are shown. Scale bars, 10 μm. The graph shows the results of the immunostaining analysis.
(TIF)

S7 Fig Dosage of X-transcripts. (**A**) The amounts of transcripts from autosomes and X chromosomes. Graphs show TPMs and SD of the amounts of transcripts from autosomes (left) and X chromosomes (right) in the cell type indicated. (**B**) X/A ratio during oogenesis in culture. The graph shows the X/A ratio in the cell type with a different set of sex chromosomes. (**C**) Relative values of X/A ratio between XX and XO. (**D**) DEGs between XX and XO oocytes. The list shows genes whose expression was 2-times higher or lower in XX oocytes compared to XO oocytes. The numbers in the heatmap are Log2(XX/XO). (**E**) DEGs between XX and XY oocytes. The list shows genes whose expression was 2-times higher in XX oocytes compared to XY oocytes. (**F**) Venn diagram of the DEGs.
(TIF)

S8 Fig Expression of Y-linked genes in XY oocytes. The expression data was extracted from the transcriptome analysis (in this study) and Sakashita et al. [18]. The values indicate the TPM of each gene at the stage indicated.
(TIF)

S9 Fig Generation of *Eif2s3y*-transgenic ESC lines. (**A**) Structure of the *Eif2s3y* or *Eif2s3x*-transgene. *Eif2s3y* or *Eif2s3x* is driven by a c-kit promoter. (**B**) Expression level of exogenous *Eif2s3y* in the transgenic ESC lines. The transcript of *Eif2s3y* was detected by Q-PCR. The graph shows the ΔCt value and SD of *Eif2s3y* referenced by *Rplp0*. Eif2s3y-F, Eif2s3y-R, Rplp0-F and Rplp0-R primers were used for this study. (**C**) Expression level of exogenous *Eif2s3x* or *Eif2s3y* in the transgenic ESC lines. The transcript of *Eif2s3x* or *Eif2s3y* was detected by Q-PCR. The graph shows the ΔCt value and SD of *Eif2s3x* or *Eif2s3y* referenced by *Rplp0*. Eif2s3x3End Fw4, PB cKit 3'UTR Rv1, Rplp0-F and Rplp0-R primers were used for this study (S1 Table).
(TIF)

S10 Fig Oocyte induction from *Eif2s3y*-KO XY ESCs. (**A**) Deletion of the *Eif2s3y* gene by Cas9. gRNAs for deletion of exons of the *Eif2s3y* gene and primers for detection of the deletion are shown. The gRNA sequences used in this study are listed in S1 Table. The image shows PCR results using Eif2s3y-gen-F and Eif2s3y-gen-R primers. (**B**) Oocyte differentiation from *Eif2s3y*-KO XY ESCs in culture. Images are rOvaries at the days indicated. Note that SC-positive cells can be observed at agg-11. Scale bars, 200 μm. (**C**) The number of SC-positive cells in the rOvaries at agg-11. Note that the number of SC-positive cells at agg-11 were significantly higher (*P<0.05, *t*-test) in the rOvaries with germ cells derived from *Eif2s3y*-KO XY ESCs (K39), compared with XY ESCs (N2).
(TIF)

S11 Fig Distinct involvement of 3’UTR of *Eif2s3x* and *Eif2s3y* in gene expression. (**A**) Genomic structure of *Eif2s3x* and *Eif2s3y*. Note that the length of 3’UTR of *Eif2s3x* is longer than that of *Eif2s3y*. (**B**) Schematic diagrams of the reporter constructs. The mCherry gene with the 3’UTR of either *Eif2s3x* or *Eif2s3y* is driven by a c-kit promoter. (**C**) FACS analysis and images of ESC lines harboring the mCherry-reporter construct. FACS analysis provided a comparison of mCHERRY expression between transgenic ESC lines (red) and a non-transgenic ESC line (blue). Images below the FACS analysis show bright field (BF) and fluorescence images of mCHERRY (mCherry) in ESC lines. The copy numbers integrated in each ESC line are shown in brackets. Scale bars, 100 μm. (**D**) Correlation of mCHERRY intensity and the copy number integrated. Red and blue dots show ES clones harboring *Eif2s3x* or *Eif2s3y*. (**E**) Correlation of mCherry mRNA expression and the copy number integrated. The relative mRNA expression is the ΔCt value of *mCherry* referenced by *Rplp0*. (**F**) Correlation of mCHERRY intensity and mCherry mRNA expression. The primer sequences used in this study are listed in S1 Table.
(TIF)

S1 Table Oligonucleotide sequences for primers and gRNAs used in this study. (XLSX)

S2 Table Antibodies used in this study. (XLSX)
