# Supplementary figures and images for: Germ cell-intrinsic effects of sex chromosomes on early oocyte differentiation in mice
Source: PLoS Genet. 2020 Mar 26;16(3):e1008676. doi: 10.1371/journal.pgen.1008676 (PMC7138321; doi:10.1371/journal.pgen.1008676)

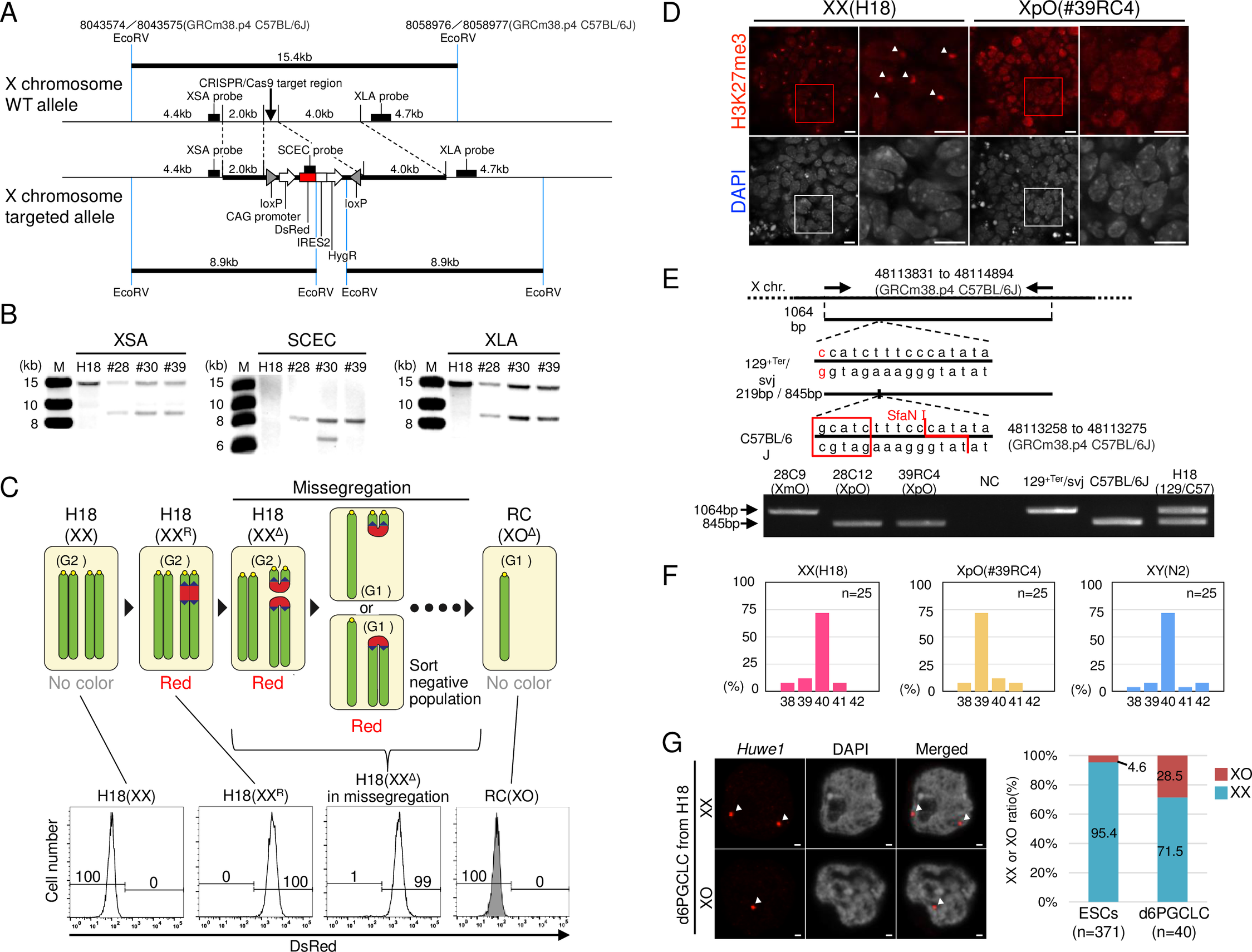

Supplement: S1 Fig — (A) Targeted integration of an elimination cassette to the X chromosome. The elimination cassette containing the DsRed gene floxed with inverted loxP sequences was introduced into the X chromosome locus. The probes and restriction enzymes for Southern blot analysis are shown. (B) Southern blot analyses using the probe described in (A). Note that the vector was properly integrated in the locus in clone #28 and #39. M, size marker. (C) Schematic diagram of elimination of the X chromosome via Cre-loxP recombination and FACS analysis. The elimination process can be monitored by expression of DsRed. FACS analysis shows DsRed expression at the elimination process. (D) Detection of the inactive X chromosome. Images show the immunofluorescence analysis of H3K27me3 (red) in EpiLCs after 1 day of aggregation culture. Arrowheads indicate punctuate staining of H3K27me3 representing the inactive X chromosome. (E) PCR analysis to distinguish the parental X chromosome. There is a polymorphism that is sensitive to SfaNI in the genome of C57BL/6J. The region can be amplified by the primers (arrows). The image below the diagram is a gel-electrophoresis of the PCR product after digestion of the enzyme. Scale bars, 10 μm. (F) Karyotype of the ESC clones. The X-axis indicates the number of chromosomes. The number of nuclei counted is shown in each graph. (G) Loss of the X chromosome in PGCLC induction. Representative images of DNA-FISH analysis of PGCLCs at day 6 of induction from BVSC H18 ESCs (left) and the quantification of the analysis (right) are shown. Scale bars, 1 μm. (TIF) [file pgen.1008676.s001.tif]

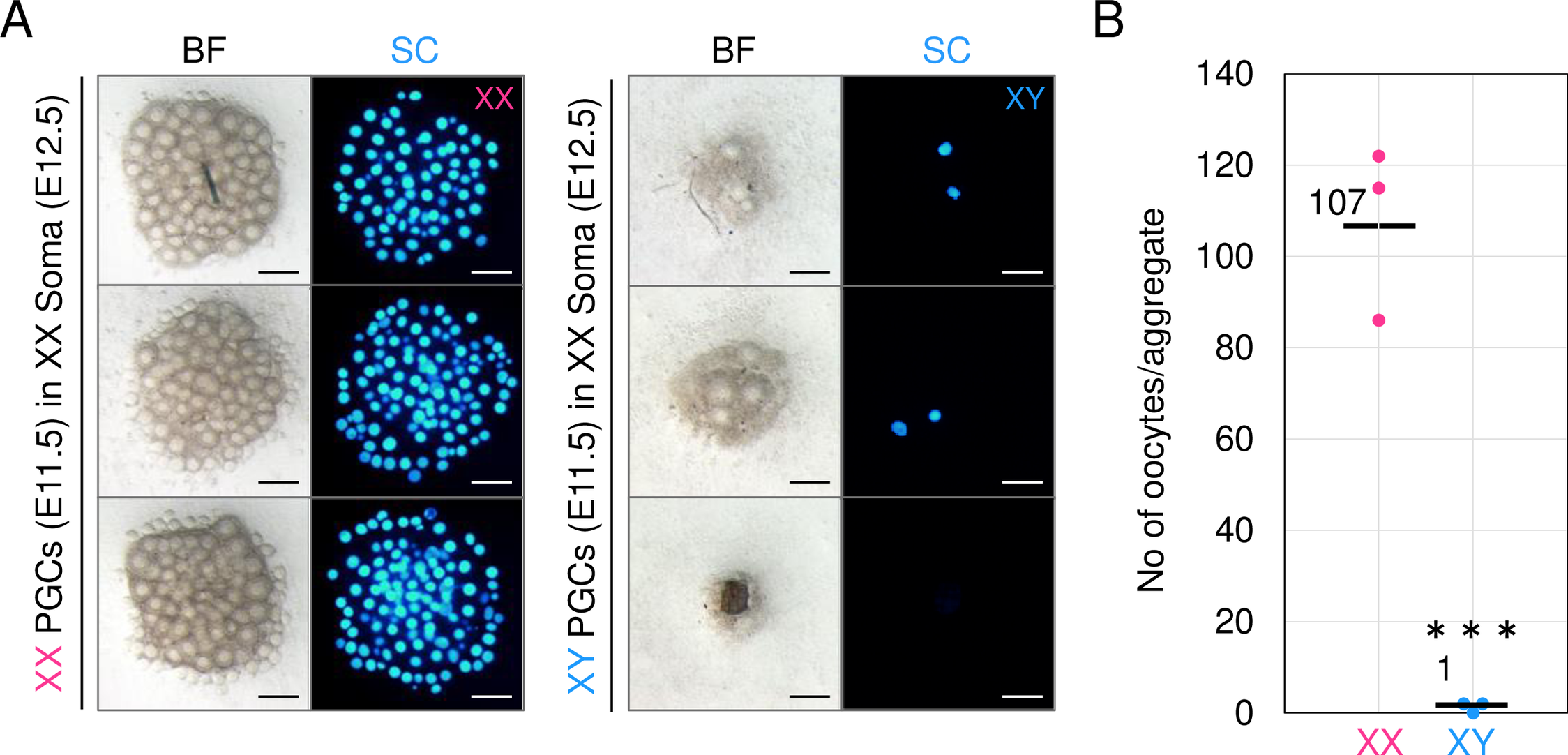

Supplement: S2 Fig — (A) Oocyte differentiation from XX and XY PGCs of E11.5 embryos. The PGCs were reaggregated with gonadal somatic cells of E12.5 female embryos. Note that the SC transgene was present in E11.5 PGCs, but not in E12.5 embryos. Scale bars, 200 μm. (B) The number of oocytes formed in culture. Each dot indicates the number of oocytes formed in one rOvary. The numbers in the graph indicate the average number of oocytes formed in each genotype. P values were calculated by t-test. ***P<0.001. (TIF) [file pgen.1008676.s002.tif]

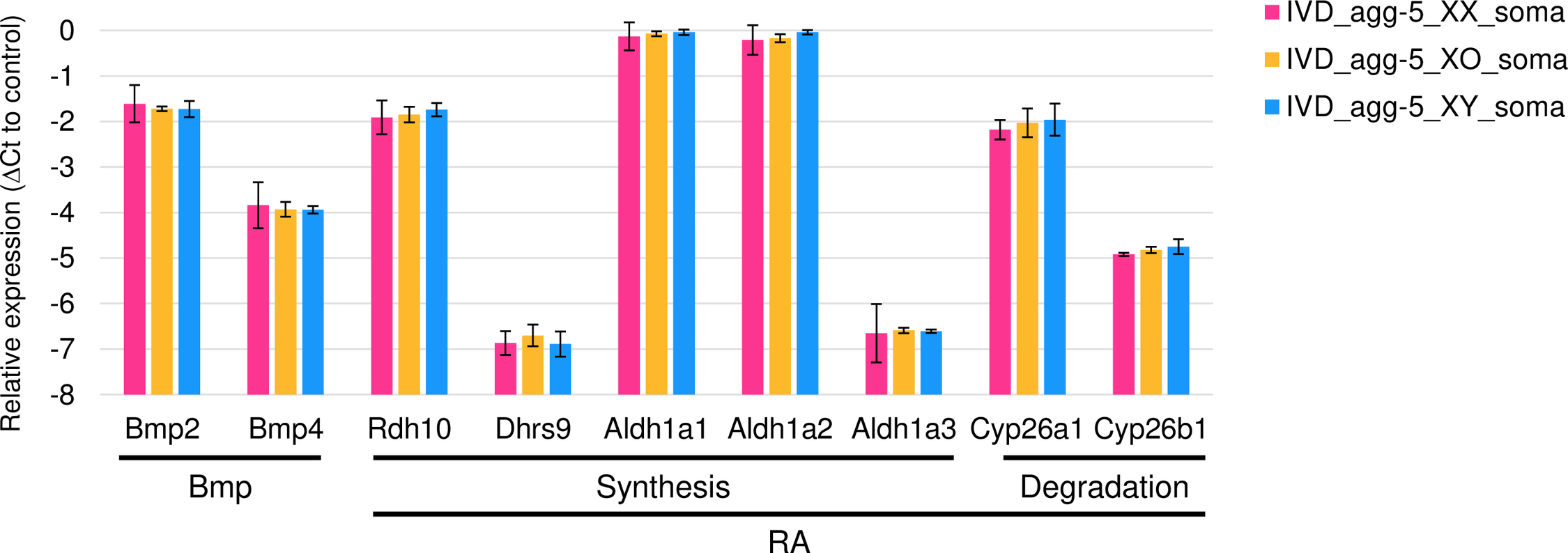

Supplement: S3 Fig — The graph shows the expression of genes related to BMP and RA signaling in the somatic cells. Somatic cells were purified from rOvaries harboring XX, XO or XY oocytes. The Y-axis shows the ΔCt value of each gene relative to the average for Rplp0 and Ppia. The primer sequences used in this study are listed in S1 Table. (TIF) [file pgen.1008676.s003.tif]

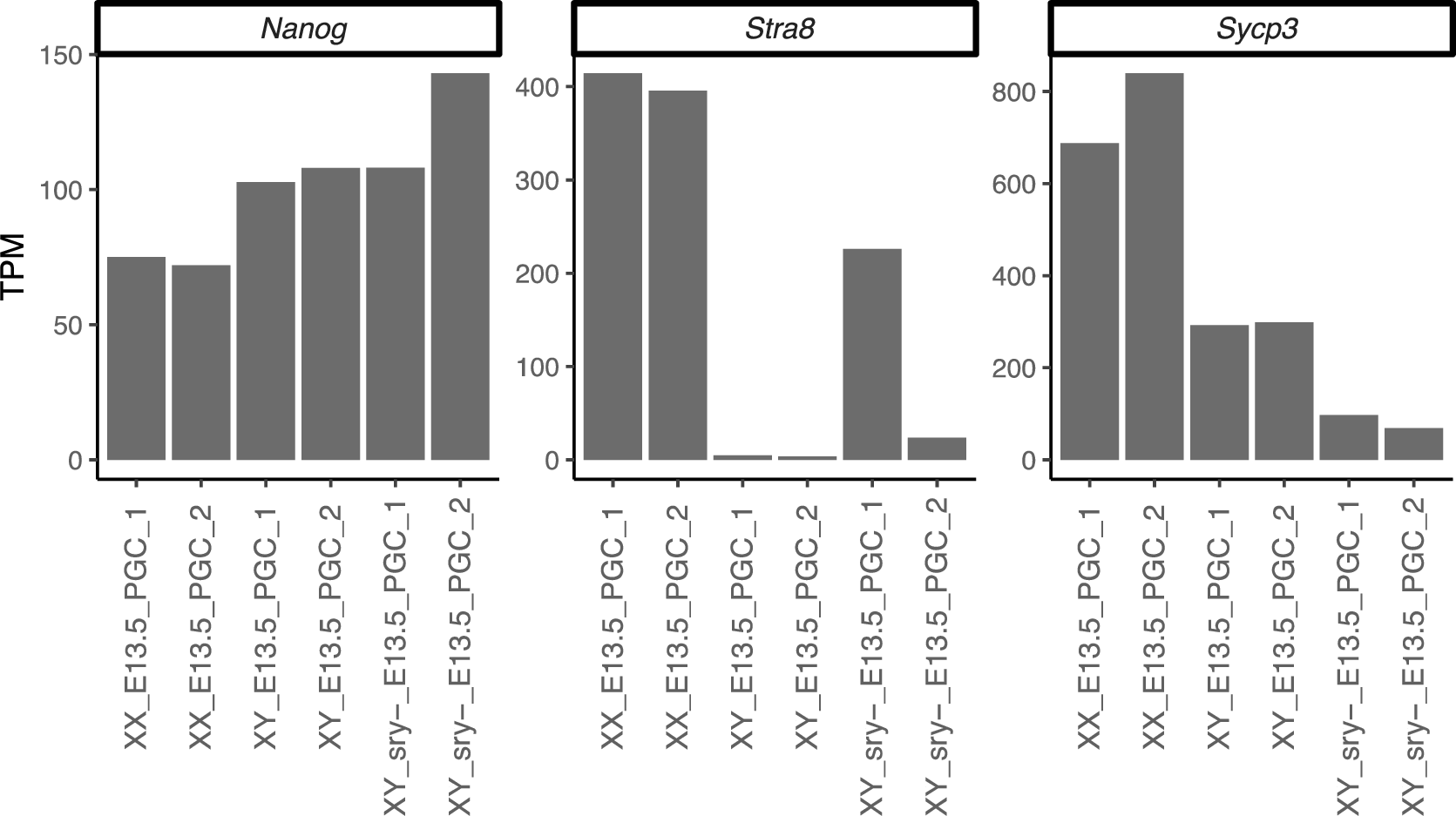

Supplement: S4 Fig — The graph shows the expression of Nanog, Stra8 and Sycp3 in XX and XYΔSry PGCs at E13.5. The expression profile was obtained from Sakashita et al. [18] (TIF) [file pgen.1008676.s004.tif]

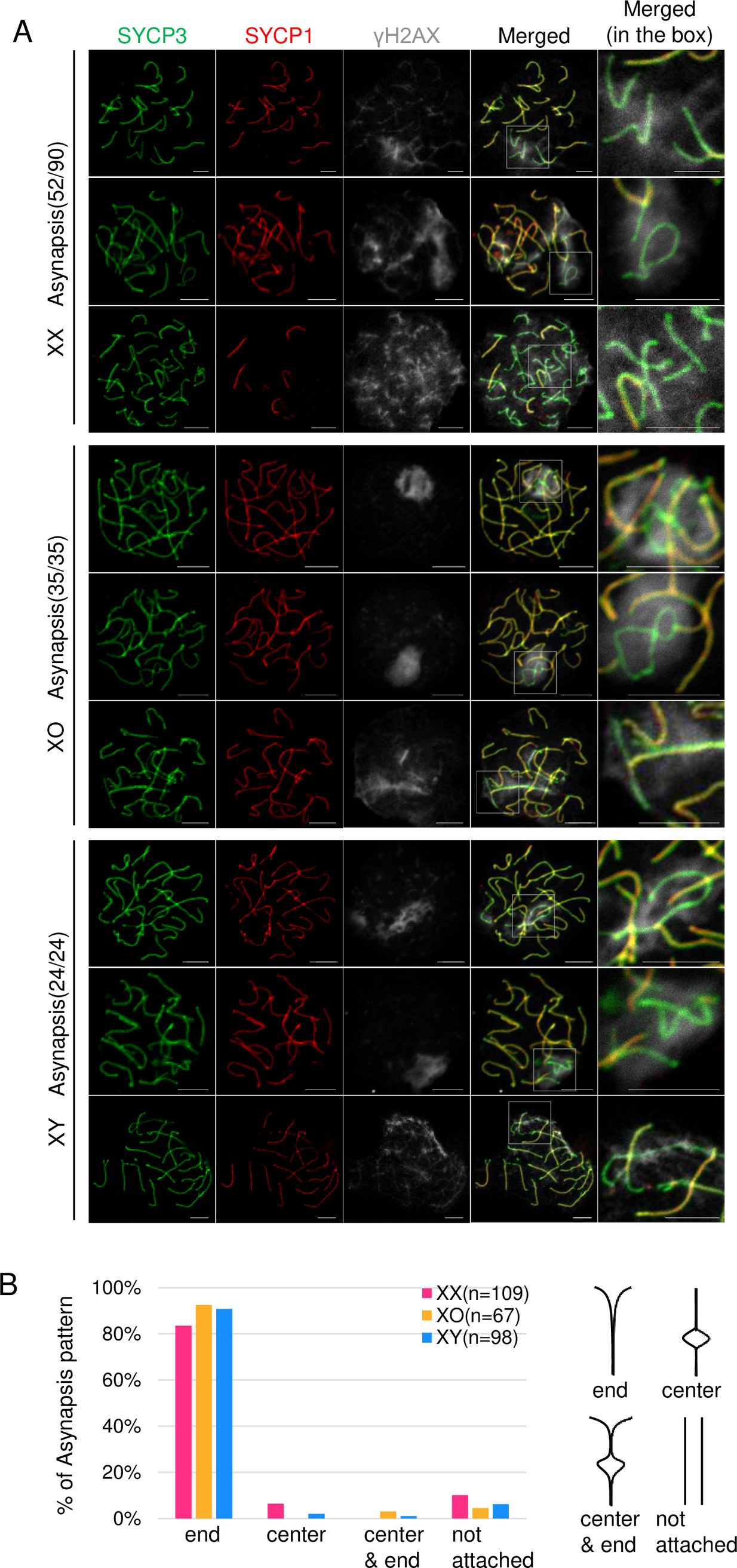

Supplement: S5 Fig — (A) Accumulation of γH2AX in the mispaired region. Three representative immunofluorescent images of SYCP3 (green), SYCP1(red), and γH2AX (white) and their merged images in XX, XO and XY oocytes are shown. The box in the merged image is shown on the right image. Note that the asynapsis regions, which are stained by SYCP3 but not SYCP1, are covered by γH2AX. (B) Pattern of autosomal asynapsis. The graph shows the percentage of each asynapsis pattern. Drawings at the right side of the graph illustrate a typical form of the chromosome in each asynapsis pattern. (TIF) [file pgen.1008676.s005.tif]

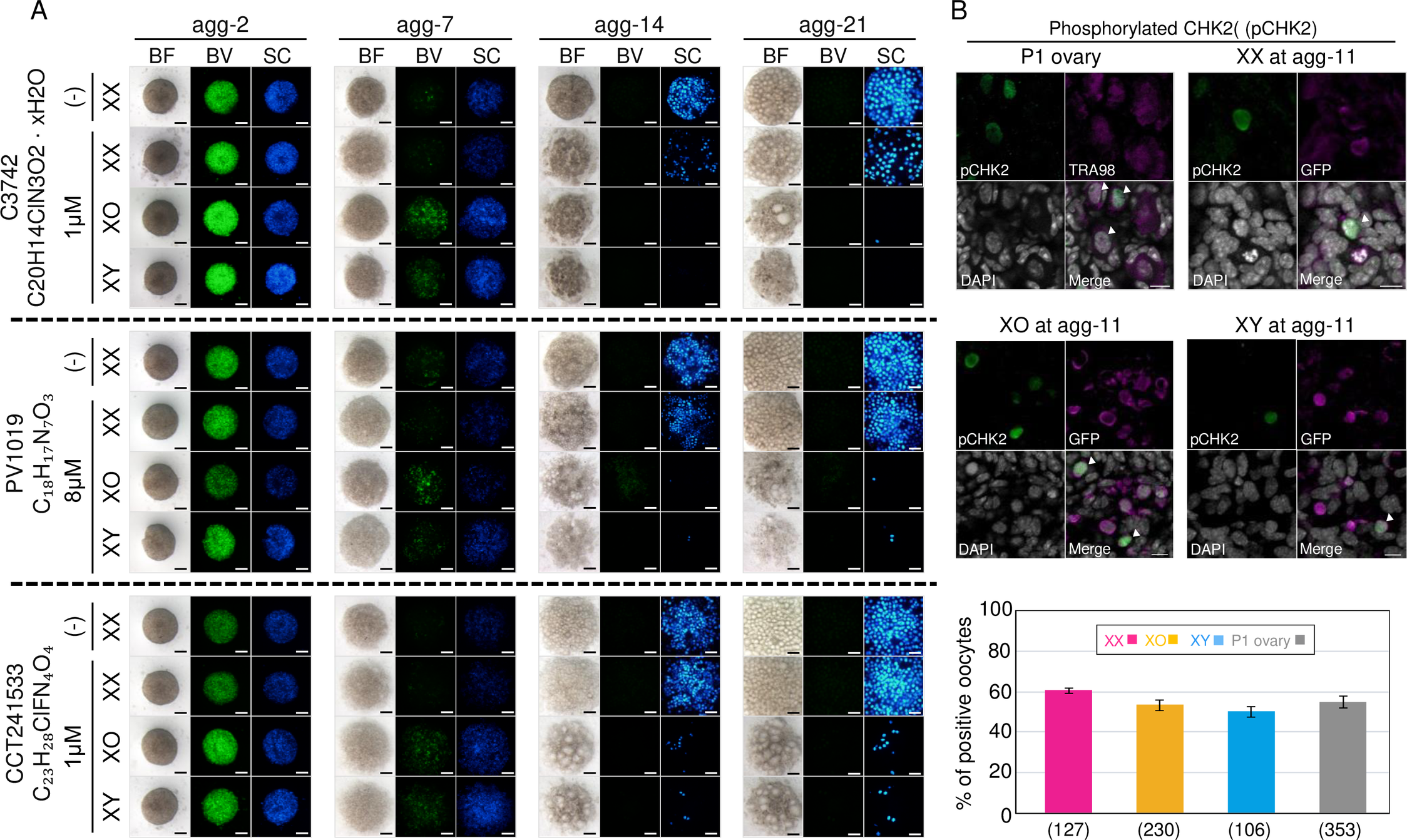

Supplement: S6 Fig — (A) Oocyte differentiation with CHK2-inhibitors. rOvaries harboring XX, XO or XY oocytes were cultured with the CHK2-inhibitors indicated at the left. Representative images at the day of culture indicated at the top are shown. Scale bars, 200 μm. (B) Immunostaining of phosphorylated CHK2 (pCHK2). Representative images of immunofluorescence analysis of pCHK2 in the P1 ovary and rOvaries harboring XX, XO or XY oocytes are shown. Scale bars, 10 μm. The graph shows the results of the immunostaining analysis. (TIF) [file pgen.1008676.s006.tif]

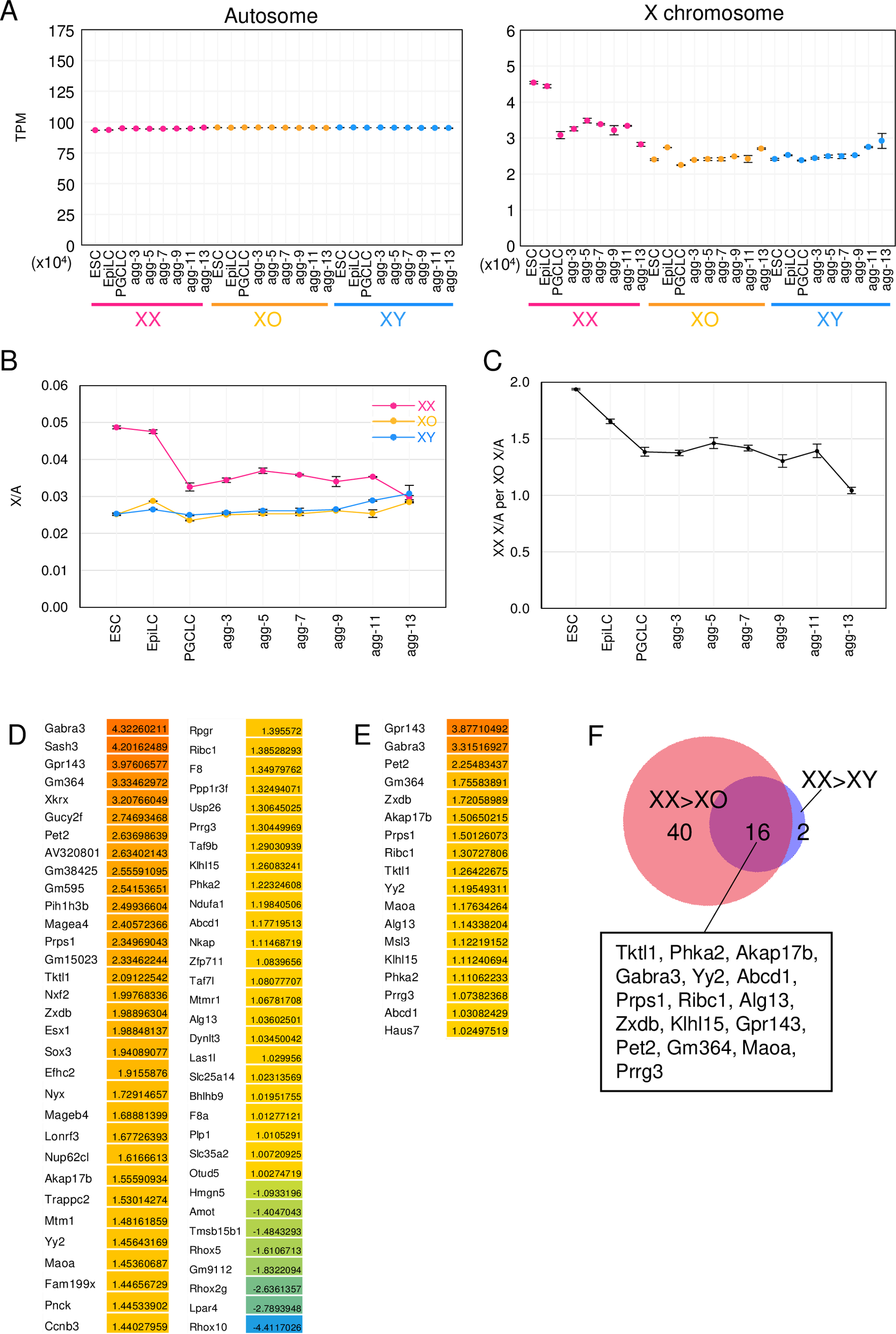

Supplement: S7 Fig — (A) The amounts of transcripts from autosomes and X chromosomes. Graphs show TPMs and SD of the amounts of transcripts from autosomes (left) and X chromosomes (right) in the cell type indicated. (B) X/A ratio during oogenesis in culture. The graph shows the X/A ratio in the cell type with a different set of sex chromosomes. (C) Relative values of X/A ratio between XX and XO. (D) DEGs between XX and XO oocytes. The list shows genes whose expression was 2-times higher or lower in XX oocytes compared to XO oocytes. The numbers in the heatmap are Log2(XX/XO). (E) DEGs between XX and XY oocytes. The list shows genes whose expression was 2-times higher in XX oocytes compared to XY oocytes. (F) Venn diagram of the DEGs. (TIF) [file pgen.1008676.s007.tif]

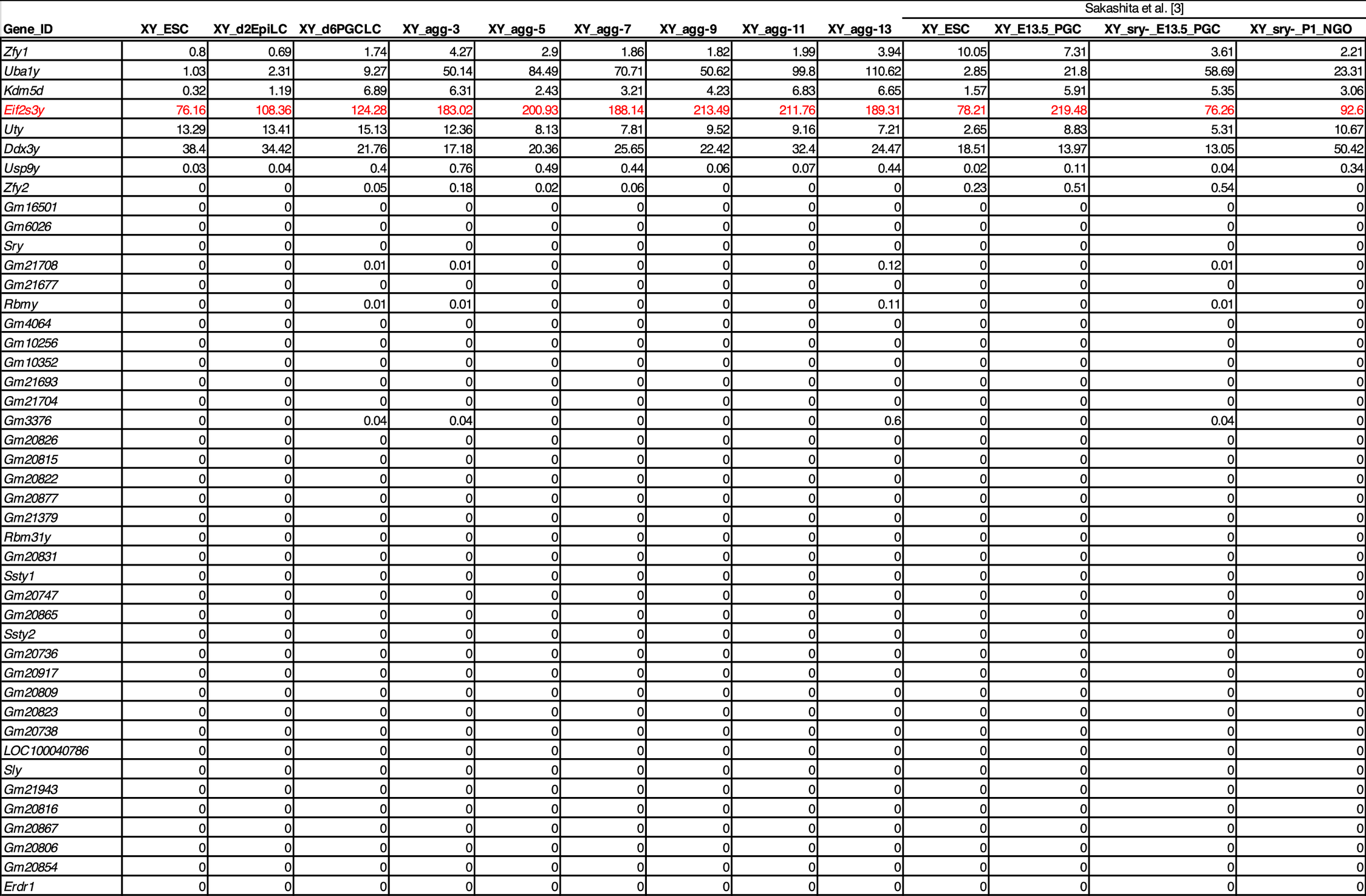

Supplement: S8 Fig — The expression data was extracted from the transcriptome analysis (in this study) and Sakashita et al. [18]. The values indicate the TPM of each gene at the stage indicated. (TIF) [file pgen.1008676.s008.tif]

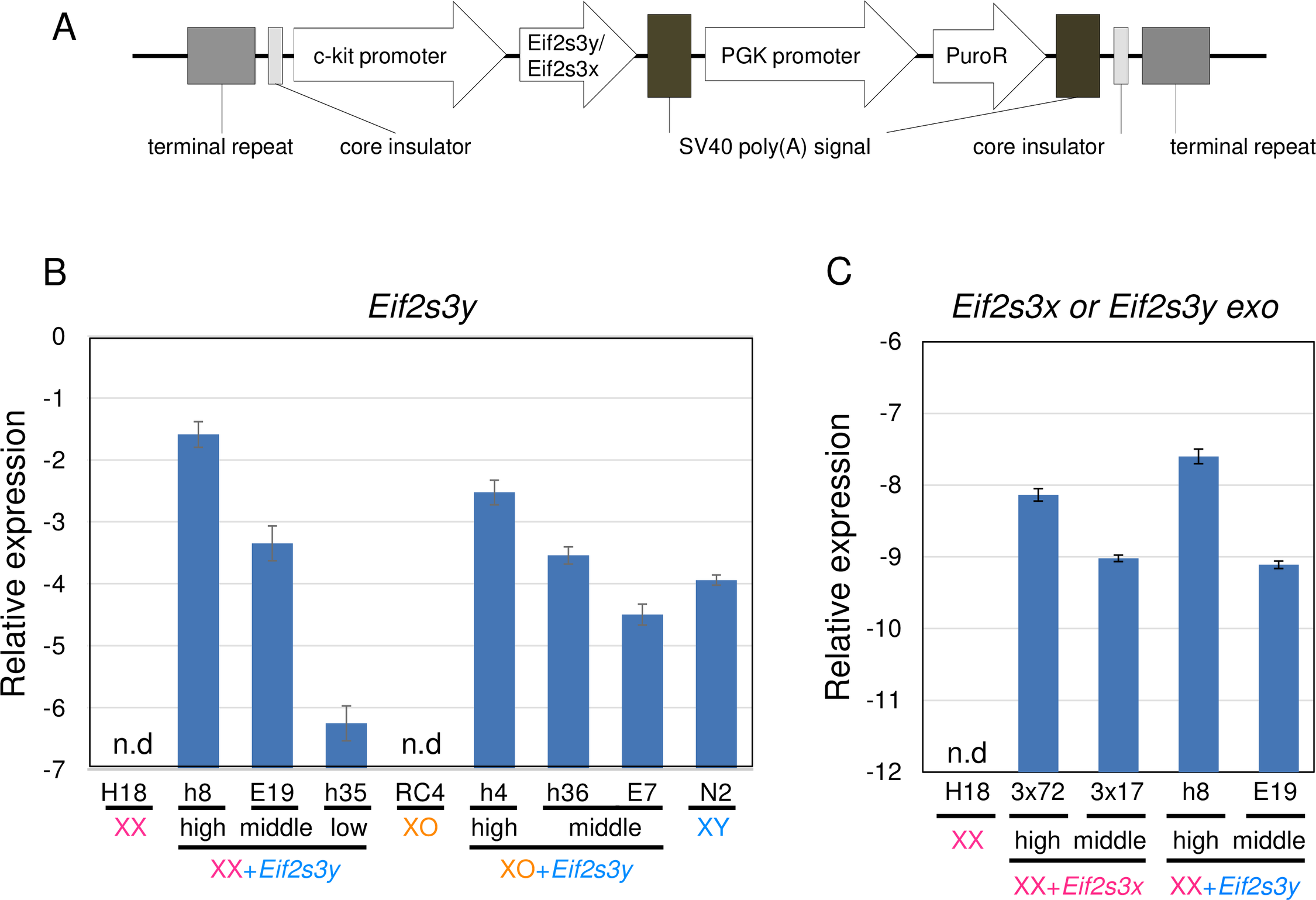

Supplement: S9 Fig — (A) Structure of the Eif2s3y or Eif2s3x-transgene. Eif2s3y or Eif2s3x is driven by a c-kit promoter. (B) Expression level of exogenous Eif2s3y in the transgenic ESC lines. The transcript of Eif2s3y was detected by Q-PCR. The graph shows the ΔCt value and SD of Eif2s3y referenced by Rplp0. Eif2s3y-F, Eif2s3y-R, Rplp0-F and Rplp0-R primers were used for this study. (C) Expression level of exogenous Eif2s3x or Eif2s3y in the transgenic ESC lines. The transcript of Eif2s3x or Eif2s3y was detected by Q-PCR. The graph shows the ΔCt value and SD of Eif2s3x or Eif2s3y referenced by Rplp0. Eif2s3x3End Fw4, PB cKit 3'UTR Rv1, Rplp0-F and Rplp0-R primers were used for this study (S1 Table). (TIF) [file pgen.1008676.s009.tif]

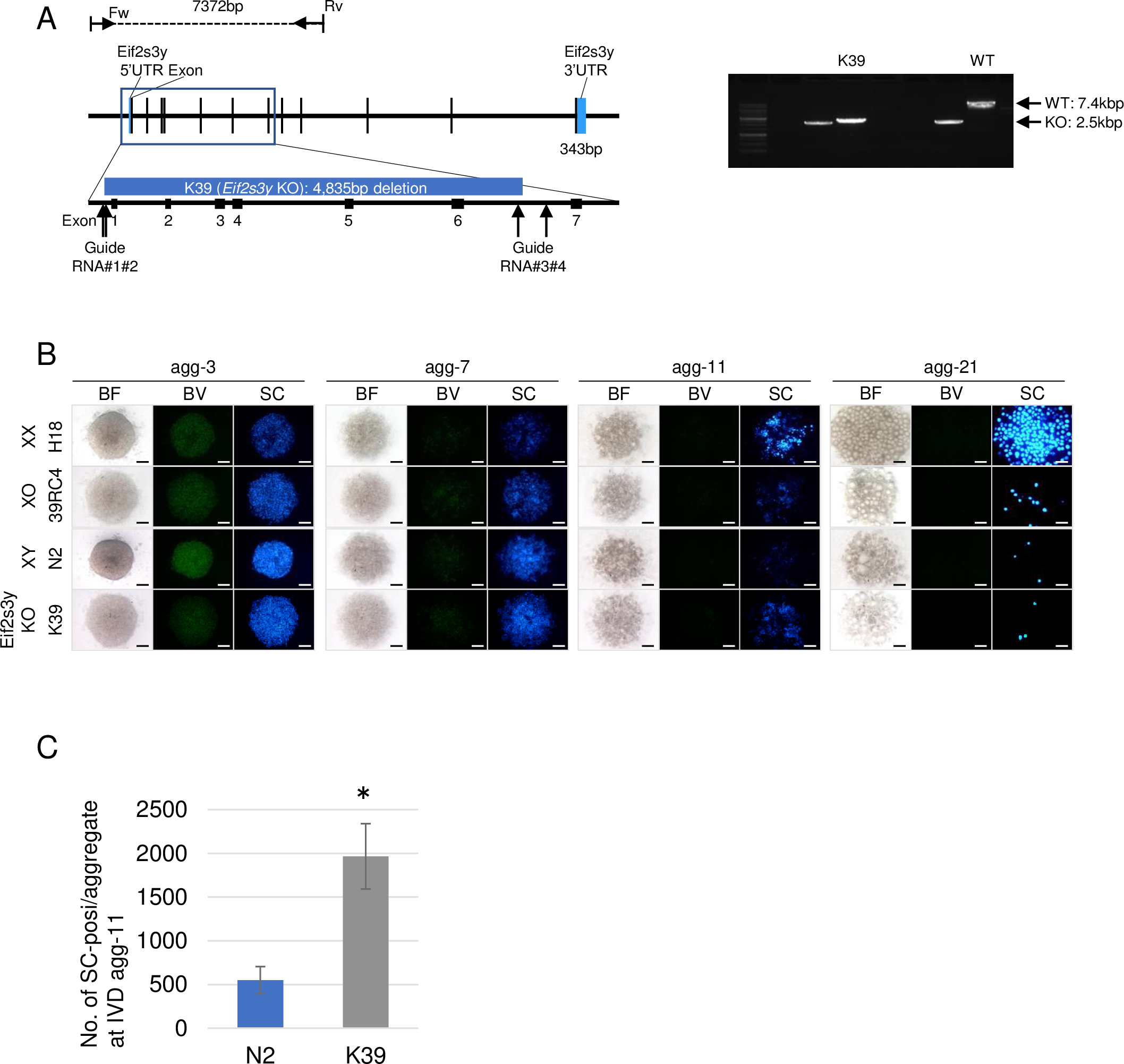

Supplement: S10 Fig — (A) Deletion of the Eif2s3y gene by Cas9. gRNAs for deletion of exons of the Eif2s3y gene and primers for detection of the deletion are shown. The gRNA sequences used in this study are listed in S1 Table. The image shows PCR results using Eif2s3y-gen-F and Eif2s3y-gen-R primers. (B) Oocyte differentiation from Eif2s3y-KO XY ESCs in culture. Images are rOvaries at the days indicated. Note that SC-positive cells can be observed at agg-11. Scale bars, 200 μm. (C) The number of SC-positive cells in the rOvaries at agg-11. Note that the number of SC-positive cells at agg-11 were significantly higher (*P<0.05, t-test) in the rOvaries with germ cells derived from Eif2s3y-KO XY ESCs (K39), compared with XY ESCs (N2). (TIF) [file pgen.1008676.s010.tif]

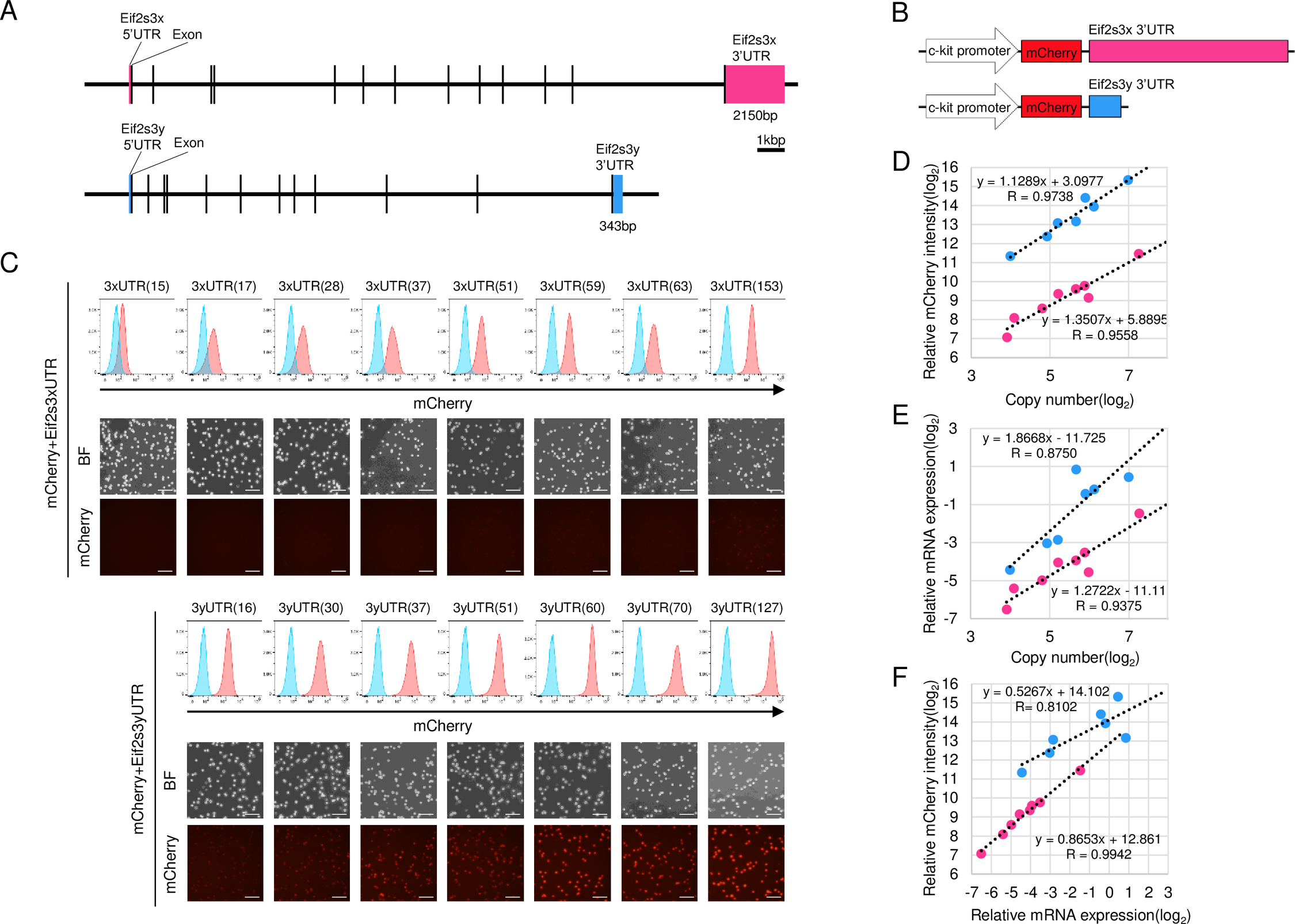

Supplement: S11 Fig — (A) Genomic structure of Eif2s3x and Eif2s3y. Note that the length of 3’UTR of Eif2s3x is longer than that of Eif2s3y. (B) Schematic diagrams of the reporter constructs. The mCherry gene with the 3’UTR of either Eif2s3x or Eif2s3y is driven by a c-kit promoter. (C) FACS analysis and images of ESC lines harboring the mCherry-reporter construct. FACS analysis provided a comparison of mCHERRY expression between transgenic ESC lines (red) and a non-transgenic ESC line (blue). Images below the FACS analysis show bright field (BF) and fluorescence images of mCHERRY (mCherry) in ESC lines. The copy numbers integrated in each ESC line are shown in brackets. Scale bars, 100 μm. (D) Correlation of mCHERRY intensity and the copy number integrated. Red and blue dots show ES clones harboring Eif2s3x or Eif2s3y. (E) Correlation of mCherry mRNA expression and the copy number integrated. The relative mRNA expression is the ΔCt value of mCherry referenced by Rplp0. (F) Correlation of mCHERRY intensity and mCherry mRNA expression. The primer sequences used in this study are listed in S1 Table. (TIF) [file pgen.1008676.s011.tif]
